# Seasonal and regional divergence in snow-regulated early-season vegetation productivity on the Tibetan Plateau

**DOI:** 10.3389/fpls.2026.1944350

**Published:** 2026-09-30

**Authors:** Qianqian Ma, Xiangyi Li, Ji Liu, Qilong Tian, Zhaobin Mu

**Affiliations:** 1 College of Ecology and Environment, Key Laboratory of Oasis Ecology of Education Ministry, Xinjiang University, Urumqi, China; 2 Xinjiang Key Laboratory of Desert Plant Roots Ecology and Vegetation Restoration, Xinjiang Institute of Ecology and Geography, Chinese Academy of Sciences, Urumqi, China; 3 State Key Laboratory of Ecological Safety and Sustainable Development in Arid Lands, Urumqi, China; 4 Cele National Station of Observation and Research for Desert Grassland Ecosystems, Urumqi, China; 5 State Key Laboratory of Loess Science, Institute of Earth Environment, Chinese Academy of Sciences, Xi’an, China

**Keywords:** climate change, productivity, snow depth, soil moisture, vegetation phenology

## Abstract

**Introduction:**

Seasonal snow significantly regulates vegetation productivity on the Tibetan Plateau (TP), but its pathways and seasonal effects remain unclear.

**Methods:**

Using multi-source remote sensing datasets and long-term field observations, this study employed a structural equation model to disentangle the dual-pathway regulatory mechanisms—specifically the soil moisture and growing-season effects—through which snow influences net primary productivity (NPP) across spring and summer on the TP.

**Results:**

Snow regulation of vegetation NPP exhibited significant seasonal and spatial differentiation. Spring (March-May) NPP was primarily influenced by the growing-season effect (GS effect), where changes in snow depth were linked to shifts in the start of the growing season (SOS), thereby modulating NPP; this relationship intensified with increasing elevation. In contrast, summer (June-August) NPP was primarily influenced by the soil moisture effect, whereby increased/decreased snow depth was associated with increased/decreased soil moisture, which may subsequently contribute to higher/lower NPP. However, this effect weakened with elevation and even became negative above 4500 meters. Spatially, the total effect of snow depth on summer NPP was positive in the semi-humid central and eastern TP, but negative in the semi-arid southwestern TP.

**Discussion:**

These seasonal and spatial variations highlight the importance of accounting for climatic and altitudinal modulation in carbon cycle models, providing vital insights into alpine ecosystem responses to ongoing climate change.

## 1 Introduction

In recent decades, the Tibetan Plateau (TP) has experienced unprecedented climate warming that has profoundly impacted its fragile ecosystem. These impacts include changes in snow depth and snow phenology, shifts in vegetation phenology and habitat distribution, and alterations in productivity (Bibi et al., 2018; Piao et al., 2019; You et al., 2020; Tang et al., 2024; Ma et al., 2025). As a critical interface between the atmosphere and the land surface, snow not only plays a crucial role in global climate feedback, but it also regulates the hydrological cycle through water storage and release processes, indirectly influencing vegetation growth and carbon sink functions (Wang et al., 2018b; Irannezhad et al., 2022; Liu et al., 2023).

Vegetation phenology is a biological factor that is highly sensitive to climate and easily observed, and it exerts strong control over ecosystem carbon sequestration (Ren et al., 2024; Yin et al., 2024; Choler et al., 2025; Wang et al., 2025). Studies have shown that warming-induced advancements in the start of the growing season (SOS) can significantly enhance vegetation productivity (Gu et al., 2022; Zheng et al., 2022; Dang et al., 2023; Bai et al., 2024; Ren et al., 2024; Li et al., 2025; Liu et al., 2025b). However, some studies suggest that earlier spring phenology may exacerbate water stress in summer or autumn, thereby limiting the accumulation of vegetation net primary productivity (NPP) (Buermann et al., 2018; Marchin et al., 2018; Zheng et al., 2020; Lian et al., 2024). Though water availability is one of the key environmental factors regulating vegetation productivity, the critical role that snow cover plays in mediating water supply has often been overlooked.

Recent simulations and integrated studies on the snow-vegetation coupling mechanism in high-latitude regions of the Northern Hemisphere have provided an important theoretical framework for understanding how changes in winter snow affect vegetation productivity (Wang et al., 2018a; Liu et al., 2023). On one hand, earlier snowmelt helps vegetation begin growing earlier, extending the time for resource acquisition and allocation, and thus increasing vegetation productivity (i.e., the “growing season effect”, GS effect) (Wang et al., 2018a; Chen et al., 2019). On the other hand, snowmelt significantly affects soil moisture, and the resulting soil moisture anomalies can persist for months, thus exerting a profound impact on vegetation productivity during the growing season (i.e., the “soil moisture effect”, SM effect), particularly in water-limited ecosystems (Wang et al., 2018a; Chen et al., 2019; Yang et al., 2019).

Previous studies have demonstrated that snow cover significantly influences vegetation growth across various regions of the Northern Hemisphere through its effects on soil moisture and vegetation phenology (Wang et al., 2018a; Chen et al., 2019; Liu et al., 2023). For instance, snow primarily exerts a positive effect on vegetation growth through the SM effect in the Midwestern United States and the Himalayas, but the dominant mechanism in Central Europe is the often-negative GS effect (Wang et al., 2018a). In temperate regions of China, delayed snowmelt has been found to significantly increase water availability in water-limited regions by reducing preseason soil moisture deficit, thereby advancing the SOS, and enhancing spring carbon uptake capacity (Chen et al., 2019). In contrast, in humid regions with adequate water availability, heavier snow cover, and later snowmelt may delay SOS and reduce gross primary productivity (GPP) in spring. These studies have elucidated the mechanisms by which snow influences vegetation growth by regulating phenology and soil moisture. However, the effects of snow on vegetation growth may differ between spring and summer, and how the interactions among these processes regulate net primary productivity (NPP) on the TP remains unclear. Notably, winter snow on the TP has generally declined across the TP over recent decades (Huang et al., 2017; Ma et al., 2023), and climate models consistently project a decrease in snowfall/rainfall ratio in future warmer winters (Wang et al., 2016; Nury et al., 2022). Therefore, quantifying the impact of winter snow changes on vegetation carbon sequestration on the TP holds significant scientific and practical importance.

To investigate the regulatory mechanisms of snow cover changes on vegetation NPP across the TP, as well as their potential seasonal differentiation, regional heterogeneity, and elevation dependence, this study sets the following objectives: (1) To retrieve the spatiotemporal dynamics of vegetation NPP and SOS on the TP using a long-term NDVI dataset (1982-2020) constructed by integrating GIMMS NDVI and MODIS NDVI data, and to validate these results across temporal and spatial dimensions with ground-based observational data. (2) To construct a structural equation model that incorporates datasets of snow depth, snowmelt date, temperature, precipitation, soil moisture, and solar radiation, systematically analyzing the pathways, directions, and intensities of snow depth’s influence on spring and summer vegetation NPP. (3) To clarify the role of elevation in modulating the ecological processes of NPP through snow depth regulation. This is the first study to reveal the seasonal and regional differences in the regulation of vegetation productivity by snow depth changes on the TP. The findings provide theoretical support and scientific evidence for improving the precision of regional carbon cycle modeling.

## 2 Materials and methods

### 2.1 Study area

The Tibetan Plateau (TP), located in the mid-latitude regions of the Northern Hemisphere, is the highest and largest plateau in the world, with an average elevation exceeding 4000 m (Kang et al., 2010; Zhang et al., 2014). The region’s topography is extremely complex, with a general pattern of lower elevations in the southeast and higher elevations in the northwest (**Figure 1A**). Influenced by altitude, topography, and monsoonal moisture, the climate of the TP exhibits a significant hydrothermal gradient from southeast to northwest: precipitation, temperature, and soil moisture gradually decrease (**Figures 1B, C, G**), while solar radiation increases (**Figure 1E**). Seasonal snowfall on the TP mainly occurs from September to February of the following year (Ma et al., 2023). The region with high snow depth (>20 cm) is predominantly found in the Golog-Nagqu high-cold shrub-meadow zone (HIB1) and the Western Sichuan-eastern Tibet montane coniferous forest zone (HIIAB1) (**Figure 1D**). Greater snow depth is generally associated with later snowmelt (**Figure 1F**), and this meltwater serves as the source for many of Asia’s major rivers (Immerzeel et al., 2010; Yao et al., 2012; Huang et al., 2017). This meltwater plays a crucial role in regulating regional vegetation growth, ecosystem services, and water resource supply. Based on temperature and moisture conditions, the TP is divided into ten major ecological geographical zones (Zheng et al., 2008), as detailed in **Table 1**.

**Figure 1:**
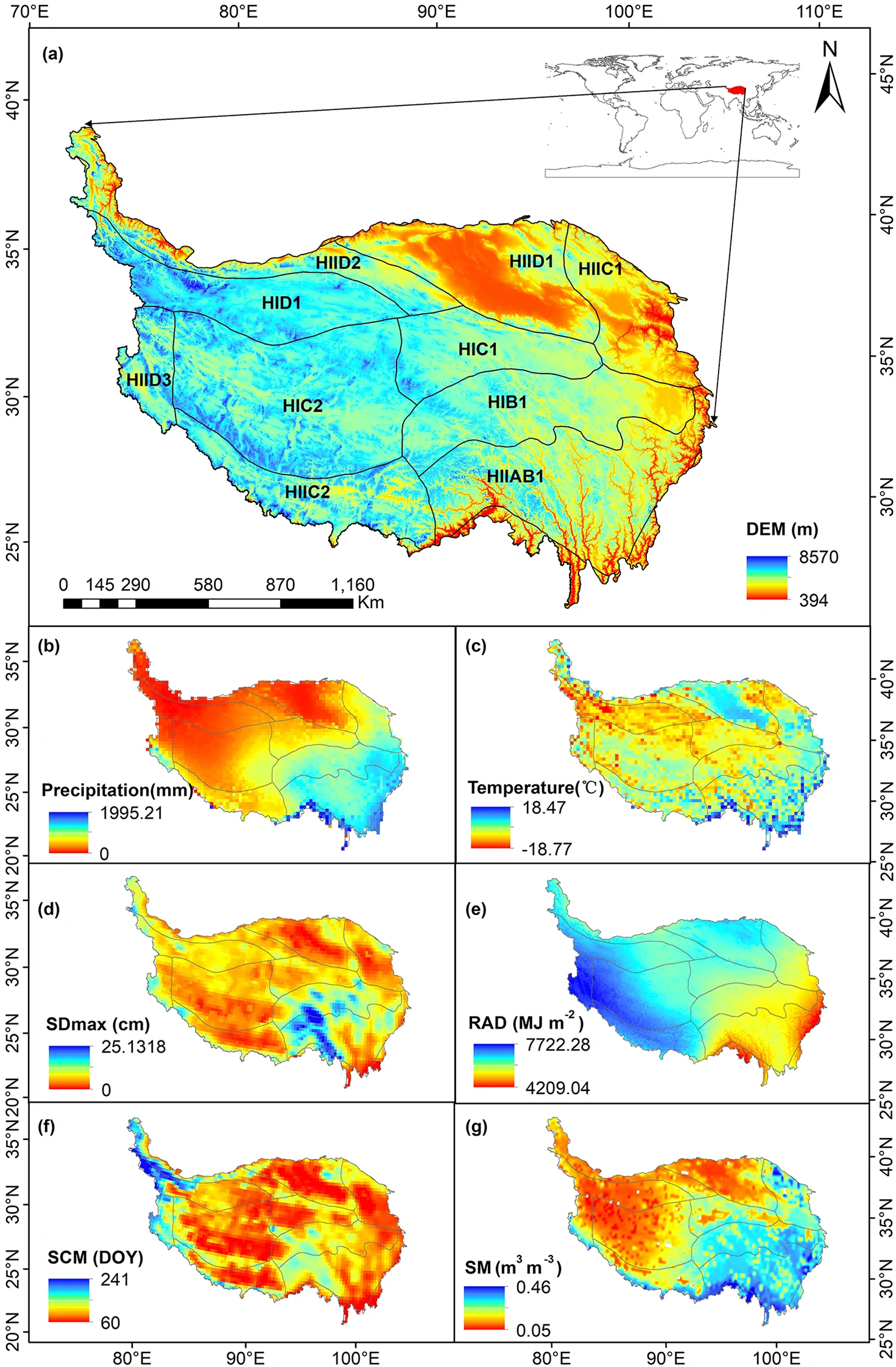
**(A)** The geographical location of the Tibetan plateau (TP). **(B)** Average annual total precipitation, **(C)** average annual temperature, **(D)** average annual maximum snow depth, **(E)** average annual total solar radiation, **(F)** average annual snowmelt date, **(G)** average annual soil moisture over the TP from 1982 to 2020.

**Table 1:** Ecogeographic regionalization codes and descriptions for the Tibetan Plateau.

Code	Temperature zone	Arid-Humid zone	Name
HIB1	Cold temperate zone	Semi-humid area	Golog-Nagqu high-cold shrub-meadow zone
HIC1		Semi-arid region	Southern Qinghai high-cold meadow steppe zone
HIC2		Semi-arid region	Qangtang high-cold steppe zone
HID1		Arid region	Kunlun high-cold desert zone
HIIC1	Mid-temperate Zone	Semi-arid region	Eastern Qinghai-Qilian montane steppe zone
HIIC2		Semi-arid region	Southern Tibet montane shrub-steppe zone
HIID1		Arid region	Qaidam montane desert zone
HIID2		Arid region	Northern slopes of Kunlun montane desert zone
HIID3		Arid region	Ngari montane desert zone
HIIAB1		Humid and semi-humid zone	Western Sichuan-eastern Tibet montane coniferous forest zone

### 2.2 Data sources

#### 2.2.1 NDVI dataset

The monthly NDVI dataset used in this study was constructed by the authors through pixel-level univariate linear regression-based models based on the GIMMS NDVI3g V1 dataset (1982-2015) and the MOD13A1 dataset (2000-2020). This integrated dataset, with a spatial resolution of 8 km, was validated for consistency (R^2^ = 0.99, P< 0.001) and has been successfully used in previous vegetation phenology studies (Ma et al., 2025).

#### 2.2.2 Meteorological dataset

Monthly mean temperature and monthly precipitation datasets were sourced from the National Earth System Science Data Center (http://www.geodata.cn). These datasets originally had a spatial resolution of 1 km (Peng et al., 2019).

Monthly total solar radiation data were estimated from monthly sunshine hours using the method described by Tong et al. (2005). The required sunshine hour data were obtained from the China Meteorological Data Sharing Service (http://data.cma.cn/). To generate a spatial raster dataset of total solar radiation, the thin plate spline interpolation method implemented in the ANUSPLIN package was applied. Elevation was incorporated as a covariate, and four spline functions were used for interpolation.

Due to the limitations of optical remote sensing data in snow depth detection and its constraints in large-scale, long-term sequence studies, we chose the relatively coarser spatial resolution daily passive microwave snow depth dataset, which is downloaded from the National Tibetan Plateau Science Data Center (TPDC, https://data.tpdc.ac.cn/home) and has a spatial resolution of 25 km (Che and Dai, 2015). This dataset provides reliable snow depth information across large spatial scales and extended time periods and has been widely used in snow cover research (Dai et al., 2012; Che et al., 2016; Chen et al., 2018; Yang et al., 2019; Ma et al., 2023). Following established methodologies (Wang and Xie, 2009), we calculated the duration days of snow cover (SCD), the onset date of snow cover (SCS), and the date of snowmelt (SCM) for each pixel. The resulting snow phenology dataset has been effectively employed in studies examining the response of snow cover to climate change (Ma et al., 2023).

The monthly soil moisture dataset for the root zone (10 to 100cm) was obtained from version 3.6a of the Global Land Evaporation Amsterdam Model (GLEAM) dataset, with a spatial resolution of 0.25°× 0.25°. This dataset was chosen due to its direct relevance to plant water availability in soil and has demonstrated high agreement with soil moisture measurements from eddy-covariance towers and soil moisture sensors in various ecosystems (Martens et al., 2017).

#### 2.2.3 Vegetation classification data

Given the extensive period of this study (1982-2020), it is important to note that vegetation may have undergone significant changes over these 40 years. Therefore, vegetation NPP was calculated using vegetation classification maps from four distinct periods (1981, 1990, 2000, and 2010). These maps, with a spatial resolution of 5km, were sourced from the China Land Cover Dataset (ChinaC) and were provided by the National Earth System Science Data Center (http://www.geodata.cn/). This dataset has demonstrated a higher accuracy than similar international products for this region (Li et al., 2017; Yang et al., 2017).

The 1981 classification map was used to calculate the NPP of vegetation for the period 1982-1989, the 1990 classification map for 1990-1999, the 2000 classification map for 2000-2009, and the 2010 classification map for 2010-2020.

#### 2.2.4 Observation data

Phenological data were obtained from the National Earth System Science Data Center (http://www.geodata.cn/), including the Haibei, Gongga, and Maoxian stations between 2006 and 2015. The ground phenology records comprised the dominant species at each site: at the Gongga station, *Pyrola calliantha Andres*, *Impatiens noli-tangere* L., and *Trillium tschonoskii Maxim*.; at the Maoxian station, *Carex* sp., *Hemerocallis citrina* Baroni., *Ophiopogon bodinieri* H. Lév., and *Rodgersia sambucifolia* Hemsl. at the Haibei station, *Elymus nutans* Griseb, *Pedicularis kansuensis* Maxim., *Gentiana* spp., and *Festuca ovina* L. For each station, the green-up dates of the dominant species were averaged to obtain the site-averaged phenology for that year, which was then used as ground validation data.

The validation of NPP estimates included both temporal and spatial components. Temporal validation assessed the model’s capability to capture long-term interannual variations in NPP, while spatial validation evaluated the agreement between simulated and observed NPP across the Tibetan Plateau. The data used for spatial validation was obtained from the field surveys conducted between July to September from 2020. A total of 203 grassland plots were investigated (**Figure 2A**), each measuring 100m×100m. In each plot, four 1m×1m quadrats were placed at the four corners and one in the center of the plot. Aboveground biomass was collected from these quadrats, oven-dried for 48 hours at 65°C, and then the dry weight was measured. Grassland NPP for each plot was calculated based on the conversion factor of 1.0g dry weight being equivalent to approximately 0.45g of carbon, and the results were expressed as g C/m^2^ (Sun et al., 2021). For the time series validation, data was obtained from biomass measurements taken at the Haibei National Field Research Station of the Alpine Grassland Ecosystem from May to September between 2003 and 2020. The data processing method for these time series measurements followed the same procedure as described for spatial validation.

**Figure 2:**
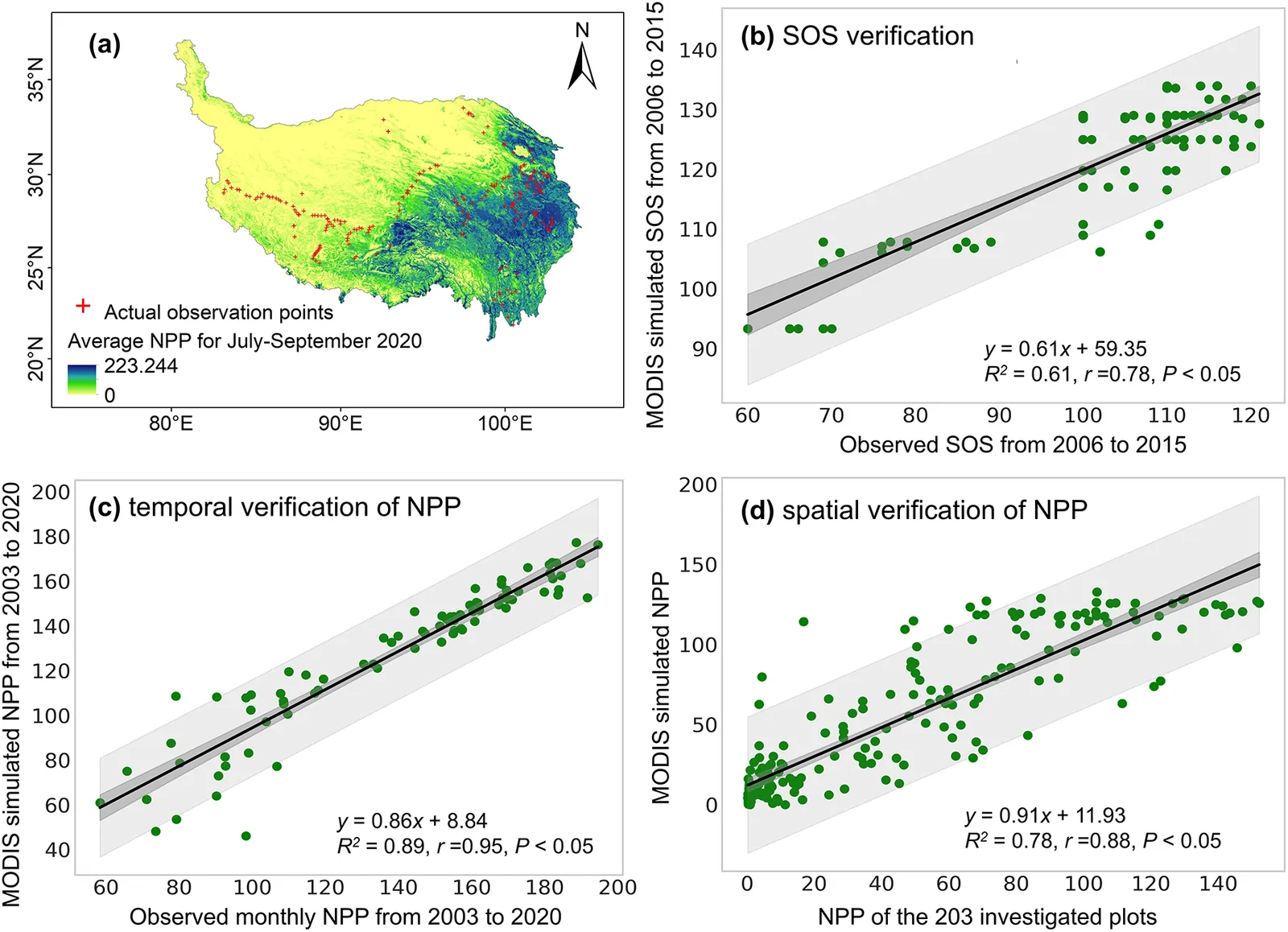
Validation results of NPP in both temporal and spatial dimensions. **(A)** Distribution map of the 203 actual biomass sampling sites. **(B)** Validation results of the start of the growing season (SOS, day of the year) based on observation data from the Haibei, Gongga, and Maoxian stations between 2006 and 2015. **(C)** Temporal validation results based on measured biomass from May to September during the period from 2003 to 2020 at the Haibei Station, compared with MODIS inversion NPP (g C m^-2^). **(D)** Spatial validation results based on 203 measured biomass (g C m^-2^) and MODIS-retrieved NPP (mean value from July to September 2020). Note: To minimize the scale discrepancy with ground data, higher-resolution MODIS simulation results were used for the fitting validation. The dark gray shadings in **(B–D)** represent the 95% confidence intervals, and the light gray shadings represent the 95% prediction intervals.

#### 2.2.5 Other data

The Digital Elevation Model (DEM) used in this study was sourced from the CGIAR Consortium for Spatial Information SRTM Digital Elevation Database (https://www.cgiar-csi.org), with a spatial resolution of 250 m. The vector range data of the TP was acquired from the Global Change Research Data Publishing and Repository (https://doi.org/10.3974/geodb.2014.01.12.V1) (Zhang et al., 2014), and the eco-geographical zone vector data was obtained from Resources and Environment Science and Data Center (https://www.resdc.cn/data.aspx?DATAID = 125). These vectors were not modified.

#### 2.2.6 Spatial data preprocessing and matching

Considering that the NDVI dataset with an 8 km spatial resolution was used for vegetation phenology extraction and CASA-based NPP estimation, all spatial datasets were standardized to the same spatial resolution and spatial reference system prior to subsequent analyses.

Datasets originally available at different spatial resolutions were resampled to an 8 km grid using the nearest-neighbor resampling method. Specifically, the temperature and precipitation datasets (1 km), vegetation classification dataset (5 km), snow depth dataset (25 km), root-zone soil moisture dataset (0.25° × 0.25°), and digital elevation model (DEM) dataset (250 m) were resampled to match the 8 km spatial resolution of the NDVI dataset. During this process, all datasets were reprojected to a common WGS84 coordinate reference system to ensure spatial consistency and pixel-level correspondence among all variables used in subsequent analyses.

The nearest-neighbor resampling method was selected because it preserves the original pixel values without introducing interpolated values or altering the inherent characteristics of the input datasets. Moreover, for categorical datasets such as vegetation classification maps, nearest-neighbor resampling is the preferred method because it maintains the original class information and avoids the generation of mixed or artificial categories during spatial aggregation.

### 2.3 Methods

#### 2.3.1 SOS and NPP inversion

The start of the growing season (SOS) from 1982 to 2020 was extracted from the extended NDVI dataset using a dynamic threshold method (Jönsson and Eklundh, 2004). Following previous research, a threshold of 0.3 was applied for SOS inversion (Ma et al., 2025). To evaluate the accuracy of the simulation results, we conducted a validation using long-term phenological observations (2006–2015) from three national monitoring stations: Haibei, Gongga, and Maoxian.

The CASA (Carnegie-Ames-Stanford Approach) model was employed to simulate the net primary productivity (NPP) of vegetation on the TP. The estimation formulas for NPP are expressed in Equations 1–3 as follows:

(1) NPP(x,t)=APAR(x,t)×ϵ(x,t)

(2) APAR(x,t)=SOL(x,t)×FPAR(x,t)×0.5

(3) $$\epsilon \left(x,t\right)=T_{\epsilon 1}\left(x,t\right)\times T_{\epsilon 2}\left(x,t\right)\times W_{\epsilon }\left(x,t\right)\times \epsilon_{max}\left(x,t\right)$$

In the equation, *APAR*(*x*, *t*) represents the absorbed photosynthetically active radiation (PAR) at pixel x in month t (MJ·m^−2^), *ϵ*(*x*, *t*) denotes the actual light use efficiency of pixel x in month t (g C·MJ^−1^), *SOL*(*x*, *t*) represents the total solar radiation at pixel x in month t (MJ·m^−2^), and *FPAR*(*x*, *t*) represents the fraction of PAR absorbed by vegetation. The constant 0.5 represents the proportion of solar radiation (with wavelengths between 0.4-0.7 μm) that can be used by vegetation. *T_ϵ_*_1_(*x*, *t*) and *T_ϵ_*_2_(*x*, *t*) represent the stress effects of low and high temperatures on light use efficiency, while *W_ϵ_*(*x*, *t*) is the water stress factor, reflecting the influence of water availability. *ϵ_max_* is the maximum light use efficiency under ideal conditions (g C/MJ). In this study, parameters such as *ϵ_max_* were adopted from Zhu et al. (2006). See **Supplementary Tables S1**–**S5** for **Supplementary Materials** for details.

#### 2.3.2 Trend analysis

We analyzed the spatiotemporal trends of snow depth, SOS, NPP, and other variables over the past four decades using the Sen’s Slope method and assessed the statistical significance of these trends using the Mann-Kendall test (Mann et al., 1945; Kendall et al., 1948).

#### 2.3.3 Structural equation modeling

Structural Equation Modeling (SEM) is a multivariate statistical analysis method primarily used to examine the relationships between complex variables, particularly for testing theoretical models and estimating causal relationships among variables (Grace, 2006; Shipley, 2016; Hair et al., 2021). In this study, we hypothesize that snow depth affects early-season (including both spring and summer) vegetation NPP by influencing vegetation phenology and soil moisture. Given that the changes in LOS on the TP are primarily driven by variation in SOS, and that SOS changes are mainly associated with snow depth and temperature (Ma et al., 2025), we took the start of the growing season (SOS), soil moisture (SM), snowmelt date, and NPP as endogenous variables, and snow depth, precipitation, temperature, and solar radiation as exogenous variables. In structural equation modeling, exogenous variables are defined as those that are not influenced by any other variables within the model. They constitute the starting point of the causal pathway and explain the variation in other variables. By contrast, endogenous variables generally represent outcome or dependent variables, the variability of which is determined by other variables in the model, including both exogenous and other endogenous variables. SEM analysis enables the comparison of both the magnitude and direction of potential correlation between two potential processes (i.e. through soil moisture changing the water supply for plant growth and through the SOS changing the length of the growing season) in winter snow depth and spring/summer vegetation productivity. Specifically, based on the standardized path coefficients obtained from the SEM models, we calculated the indirect effects of the two major pathways, including the growing-season pathway (SOS) and the soil moisture pathway (SM). Assuming multiple pathways link snow depth to NPP via SOS or SM, we initially calculated the product of the standardized path coefficients for each pathway. Then we summed up the products over all pathways to determine the impact of snow depth on vegetation NPP through either the soil moisture effect or the growing season effect. In this study, the total effect of snow depth on NPP refers to the combined contribution of the soil moisture effect and the growing-season effect, rather than the conventional SEM definition of the sum of direct and indirect effects.

To construct the SEM models, we used the R package lavaan. This is a global estimation that minimizes the difference between the observed data covariance and the model-implied covariance by estimating the model parameters through the maximum likelihood method (Grace, 2006; Shipley, 2016). To evaluate the goodness of fit between the SEM models and the observed data, we assessed multiple fit indices, including the Chi-square test (P > 0.05), root mean square error of approximation (RMSEA), comparative fit index (CFI), and standardized root mean square residual (SRMR). These indices were used to evaluate the overall agreement between the hypothesized model structure and the observed data and to identify potential model deficiencies that may require structural adjustments. Following previous studies (Wang et al., 2018a), the recommended thresholds were set as RMSEA< 0.10, CFI > 0.95, and SRMR< 0.09. Considering that SEM model evaluation should not rely on a single fit index, all models were assessed using multiple goodness-of-fit criteria. A model was considered acceptable when at least two of the three indices (RMSEA, CFI, and SRMR) satisfied the predefined criteria.

## 3 Results

### 3.1 SOS and NPP verification

To minimize scale discrepancies with ground data, higher-resolution MODIS simulation results were used for fitting validation. Ground validation data of SOS were obtained from the observation records at Haibei, Gongga, and Maoxian stations between 2006 and 2015. A regression analysis was performed using the observed values as the x-axis and the simulated values as the y-axis (**Figure 2B**). The results revealed a significant correlation between simulations and observations (r = 0.78, P< 0.05), with the coefficient of determination (R^2^) of 0.61, indicating that the simulated SOS closely matched the observed values.

The validation of NPP demonstrates a high degree of consistency between our simulated results and the measured data, both temporally and spatially (**Figures 2C, D**). For spatial validation, ground data were obtained from 203 biomass measurement plots investigated between July to September 2020 (**Figure 2A**), while simulated values were extracted from MODIS-retrieved NPP for the same period. Regression analysis revealed a strong correlation between spatially simulated and observed results (r = 0.88, P< 0.05) with R^2^ of 0.78, indicating high spatial consistency between simulations and observations (**Figure 2D**). Temporal validation, based on biomass measurements from May to September between 2003 and 2020 at the Haibei Station, showed an even stronger correlation (r = 0.95, R^2^ = 0.89, P< 0.05), indicating that the simulation accurately captured the intra-annual variations of NPP over the study period (**Figure 2C**).

### 3.2 Effect of snow depth on spring and summer NPP in the TP

Considering that this study focused on vegetation responses to snow and climate variability, only eco-geographical regions with sufficient vegetation coverage and reliable SOS/NPP retrievals were included in subsequent analyses. Regions dominated by barren land or sparse vegetation were excluded due to insufficient valid pixels.

We conducted structural equation model analysis for the entire TP and its six ecological geographical zones, identifying the optimal model for each region. The initial model included eight variables, each has 39 time-series samples spanning 1982-2020. The regional path coefficients were calculated based on the mean values of each variable within the effective pixels of the region. Effective pixels were defined as vegetated pixels that met two criteria: (1) reliable SOS estimates (60 ≤ SOS ≤ 200) and (2) complete 39-year continuous time-series records for subsequent analyses. All models passed the chi-square test (**Figure 3**; **Supplementary Figure S1**). In addition, at least two indicators from RMSEA, CFI, and SRMR met the model fit criteria. For both the entire TP and each subzone, the model in which snow depth directly affects SOS showed better fit than the model in which snow depth affects SOS through snowmelt date. Therefore, the path of SCM’s effect was removed in all models. On the entire TP, the effect of SOS on soil moisture was found to be very small and insignificant, so this path was removed. However, in different ecological zones, the effect of SOS on soil moisture was significant, so this path was retained in the subzone-specific analyses.

**Figure 3:**
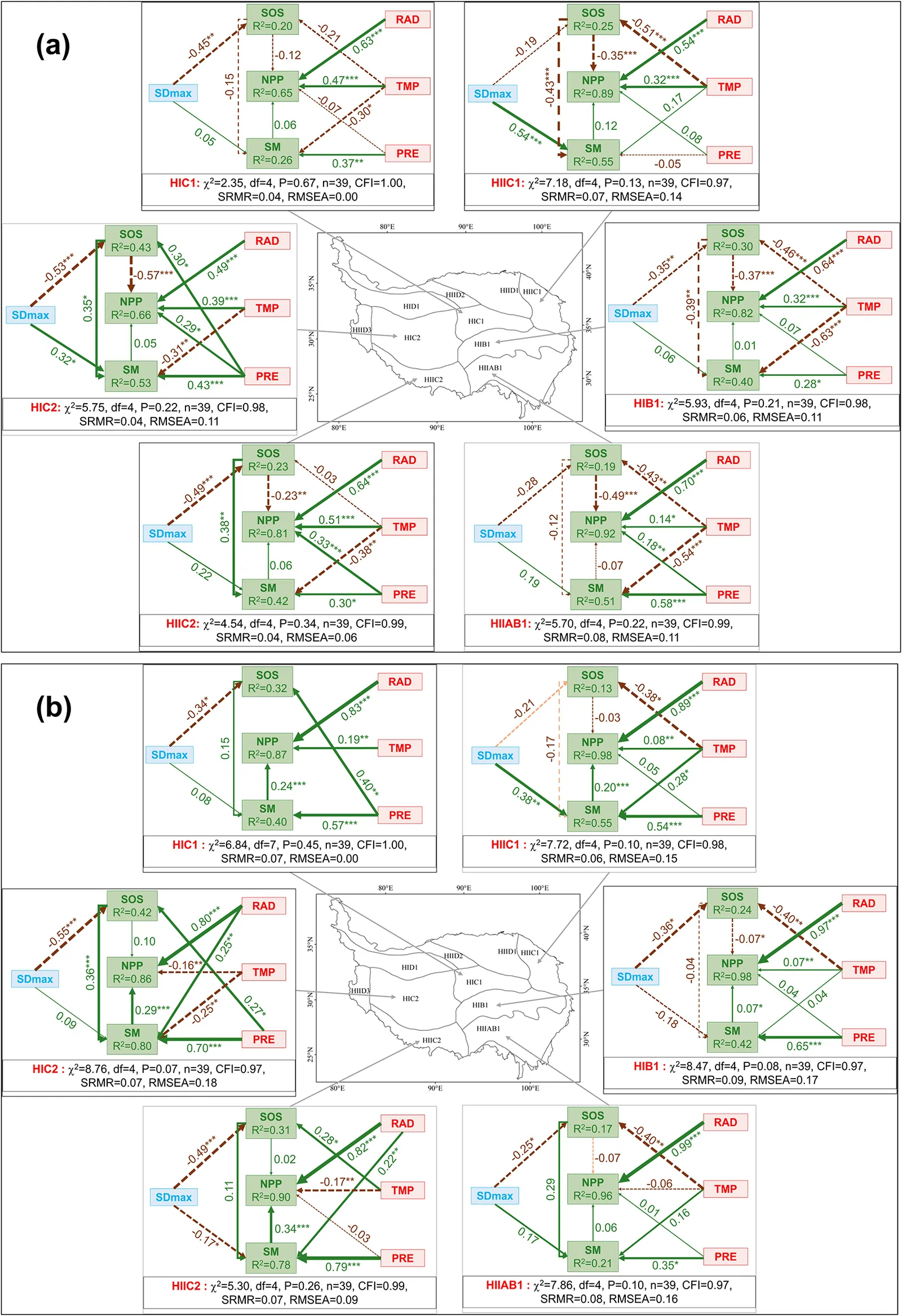
Influence pathways of snow depth on spring NPP **(A)** and summer NPP **(B)** across different ecological zones. SDmax represents snow depth, SOS represents the start of the growing season; TMP represents spring/summer temperature; RAD represents spring/summer solar radiation; SM represents spring/summer soil moisture; PRE represents spring/summer precipitation; and NPP represents spring/summer net primary productivity. All the path coefficients in the image are standardized path coefficients. The green solid lines represent positive effects, while the brown dashed lines indicate negative effects. *Indicates significance at the 0.05 level, **indicates significance at the 0.01 level, and ***indicates significance at the 0.001 level.

Since the path coefficient is analogous to the partial correlation coefficient that describes the strength and sign of the relationship between two variables, we used the spatial pattern of partial correlations between the two target variables to represent the spatial variation of the path coefficients. For example, the path coefficient describing the effect of SOS on spring NPP can be considered as their partial correlation coefficient while controlling for the effect of soil moisture, temperature, precipitation and insolation.

Across the entire TP, spring NPP was primarily influenced by the growing season effect (GS effect: 0.15), while summer NPP was significantly affected by the soil moisture effect (SM effect: -0.03) (**Supplementary Figure S1**). In spring, soil moisture mainly comes from precipitation (Path Coefficient (PC): 0.50***); thus, higher temperatures lead to reduced soil moisture (PC: -0.33*). With the arrival of summer, increased temperatures facilitate snowmelt, which significantly contributes to soil moisture (PC: -0.25*), thereby notably enhancing summer NPP (PC: 0.13***). During this process, the amount of solar radiation also contributes significantly to NPP in both spring (PC: 0.67***) and summer (PC: 0.94***).

Specifically, in the Eastern Qinghai-Qilian montane steppe zone (HIIC1), snow depth contributed significantly to soil moisture in both spring (PC: 0.54***) and summer (PC: 0.38**), but it had no significant effect on the SOS. Therefore, the “SM effect” was greater than the “GS effect” in this region (**Figures 3A, B**, **4G, H**). Over the past four decades, the increase in snow depth (**Figure 5A**) was associated with an increase in soil moisture (**Figures 5K, L**, **6G, H**), which in turn has augmented spring and summer NPP (PC: 0.12/0.20***) (**Figures 5C, D**, **6I, J**). The advancing SOS in the HIIC1 was mainly driven by the rise in temperature (PC: -0.51***) (**Figure 5E**; **Supplementary Figure S2a**), which also augmented the spring NPP (PC: -0.35***) (**Figures 5C**, **6C**).

**Figure 4:**
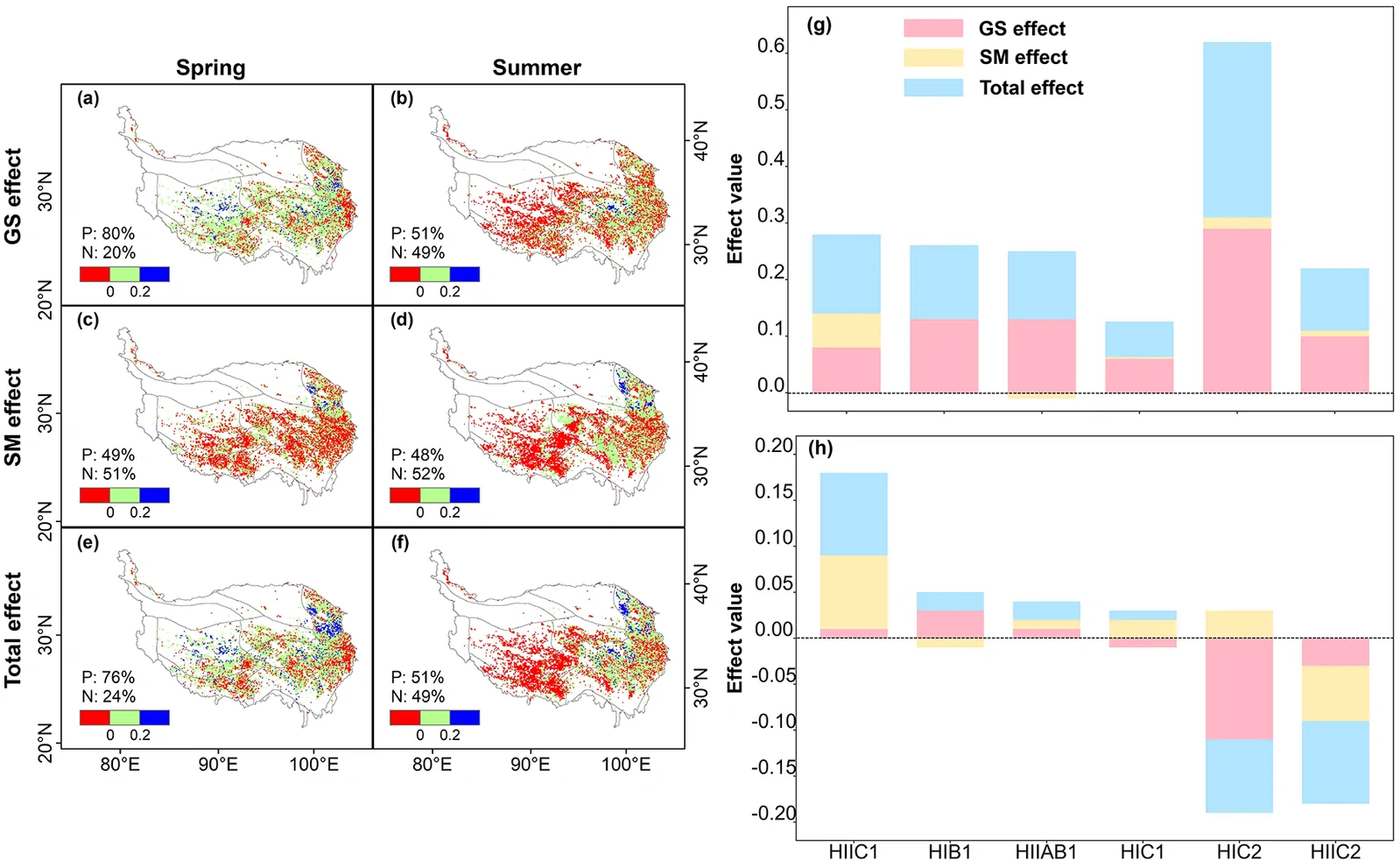
Panels **(A–F)** showed the spatial distribution patterns of growing season effects (GS effect), soil moisture effects (SM effect), and total effects in spring and summer. Panels **(G–H)** showed the comparison of growing season effect, soil moisture effect and total effect across different ecological zones during spring and summer. Note: The removal of outliers from the SOS dataset led to discontinuities in the time series of certain pixels, thereby resulting in missing values. To ensure consistent pixel correspondence for subsequent analyses, other variables were masked and extracted based on the SOS dataset.

**Figure 5:**
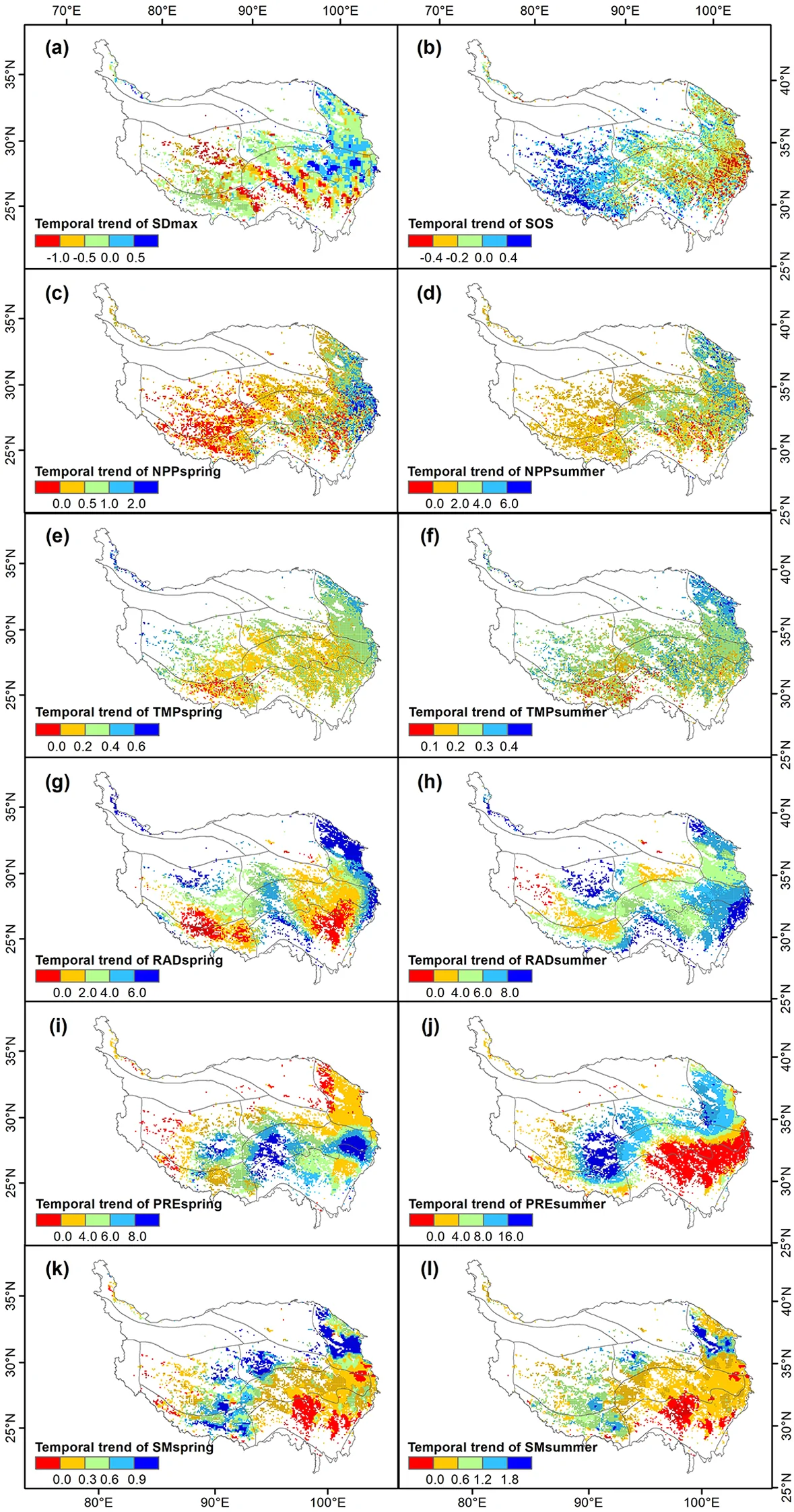
Temporal trend of **(A)** snow depth (SDmax, mm year^-1^), **(B)** start of the growing season (SOS, days year^-1^), **(C)** spring NPP (NPPspring, g C m^-2^ year^-1^), **(D)** summer NPP (NPPsummer, g C m^-2^ year^-1^), **(E)** spring temperature (TMPspring, °C decade^-1^), **(F)** summer temperature (TMPsummer, °C decade^-1^), **(G)** spring solar radiation (RADspring, MJ m^-2^ year^-1^), **(H)** summer solar radiation (RADsummer, MJ m^-2^ year^-1^), **(I)** spring precipitation (PREspring, mm decade^-1^), **(J)** summer precipitation (PREsummer, mm decade^-1^), **(K)** spring soil moisture (SMspring, dm^3^ m^-3^ year^-1^), **(l)** summer soil moisture (SMsummer, dm^3^ m^-3^ year^-1^). The removal of outliers from the SOS dataset led to discontinuities in the time series of certain pixels, thereby resulting in missing trend values. To ensure consistent pixel correspondence for subsequent analyses, other variables were masked and extracted based on the SOS dataset.

**Figure 6:**
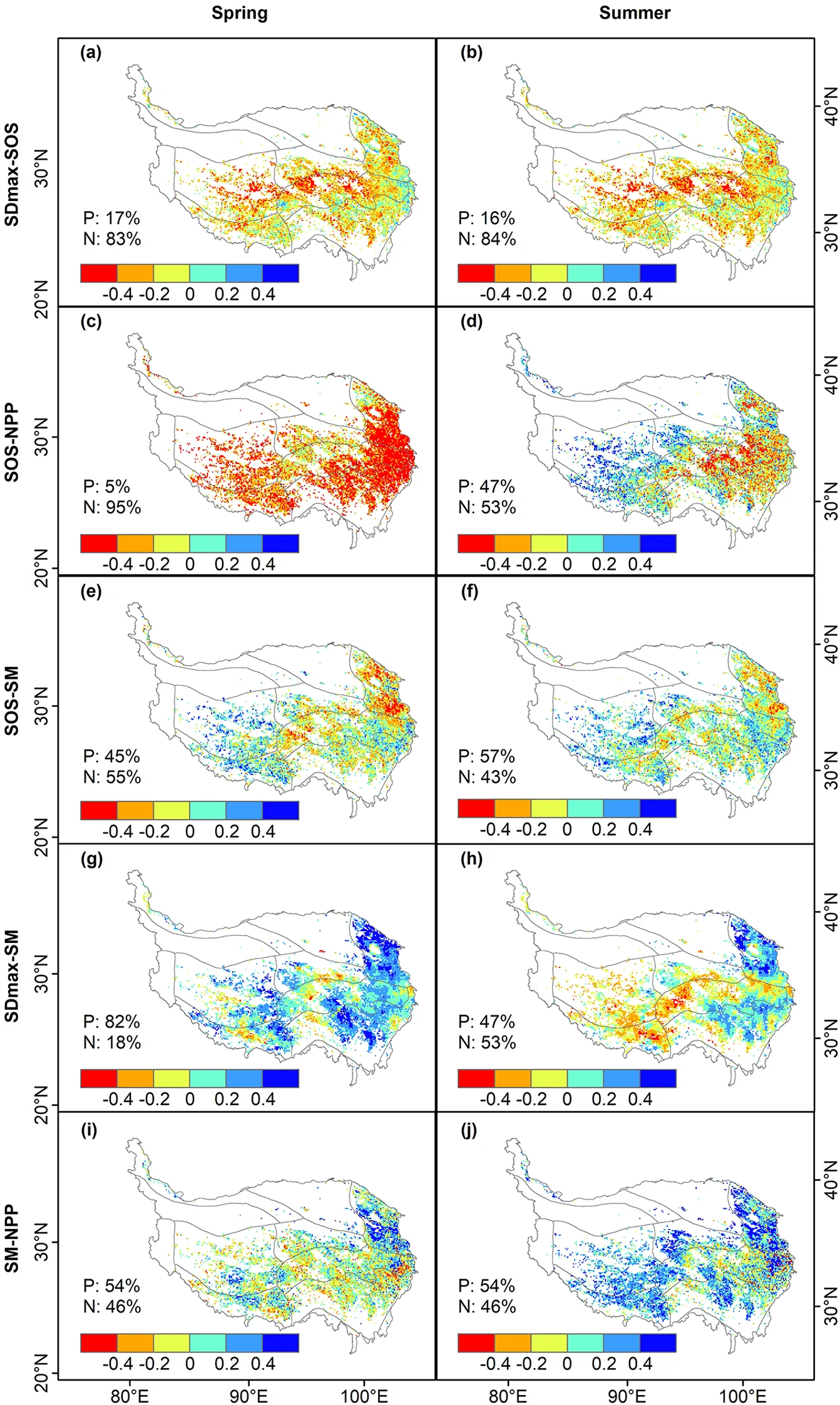
Path coefficient of snow depth regulating vegetation NPP via vegetation phenology and soil moisture. In the image, P represents a positive correlation, and N represents a negative correlation. SDmax represents snow depth, SM represents soil moisture, SOS represents the start of the growing season, and NPP represents net primary productivity. Additionally, the removal of outliers from the SOS dataset led to discontinuities in the time series of certain pixels, thereby resulting in missing values. To ensure consistent pixel-wise correspondence for subsequent analyses, other variables were masked and extracted based on the SOS dataset.

In the Golog-Nagqu high-cold shrub-meadow zone (HIB1), the “GS effect” was greater than the “SM effect” (**Figures 4G, H**). Specifically, snow depth did not contribute significantly to soil moisture in spring and summer, with the main source instead being precipitation (PC: 0.28*/0.65***). Snow depth did contribute significantly to SOS (PC: -0.35**). Over the past four decades, this region has experienced an increase in snow depth and temperature (**Figures 5A, E**), which was associated with an earlier SOS (**Figure 5B**) and an increase in spring and summer NPP (**Figures 5C, D**).

In the Western Sichuan-eastern Tibet montane coniferous forest zone (HIIAB1), the “GS effect” was greater than the “SM effect” (**Figures 4G, H**). However, the regulation of snow depth on NPP in this region was generally weak. The advancement of SOS was primarily driven by rising spring temperatures (PC: -0.43**), which subsequently enhanced spring NPP as well (PC: -0.49***). The increase in summer NPP, on the other hand, can be attributed mainly to increased solar radiation (PC: 0.99***).

In the Southern Qinghai high-cold meadow steppe zone (HIC1), the “GS effect” was greater than the “SM effect” in spring (**Figure 4G**), but weaker in summer (**Figure 4H**). Specifically, snow depth significantly affected variation in SOS (PC: -0.45**), but it did not contribute significantly to soil moisture. The main source of soil moisture in spring and summer was precipitation (PC: 0.37**/0.57***). The NPP increases in this region during spring were mainly driven by the increases in temperature (PC: 0.47**) and solar radiation (PC:0.63***). Summer NPP is mainly influenced directly by solar radiation, temperature and soil moisture, and indirectly by SOS (**Figure 3B**).

In the Qangtang high-cold steppe zone (HIC2), the “GS effect” surpassed the “SM effect”. Snow depth has the strongest regulatory effect on NPP in this region, with a generally positive effect in spring and a generally negative effect in summer (**Figures 4G, H**). Specifically, snow depth significantly affected both SOS and soil moisture in the region, with a greater impact on SOS. In addition to being influenced by snow depth (PC: -0.53***/-0.55***), SOS was also significantly affected by precipitation (PC: 0.30*/0.27*). Moreover, spring soil moisture was significantly influenced by both snow depth (PC: 0.32*) and precipitation (PC: 0.43***), while summer soil moisture was primarily driven by precipitation (0.70***). Over the past four decades, the significant reduction in snow depth (**Figure 5A**) in the HIC2 region was associated with a notable delay in the SOS (**Figure 5B**) (PC: -0.53***), which in turn has reduced the accumulation of spring NPP (**Figure 5C**) (PC: -0.57***). Moreover, the delayed SOS has alleviated summer soil moisture deficits (PC: 0.36***) and has subsequently increased summer NPP (PC: 0.29***). The spatial pattern of the path coefficients is shown in **Figure 6**; **Supplementary Figures S2**, **S3**.

In the Southern Tibet montane shrub-steppe zone (HIIC2), snow depth primarily regulates NPP through the growing season effect in spring (**Figure 4G**), while in summer, it mainly regulates NPP through the soil moisture effect (**Figure 4H**). Specifically, over the past four decades, snow depth in this region has significantly decreased (**Figure 5A**), thus leading to a delay in SOS (**Figures 5B**, **6A**). The delayed SOS subsequently reduced spring NPP (PC: -0.57***). Additionally, spring NPP was also influenced by temperature (PC: 0.51***), precipitation (PC: 0.33***), and solar radiation (PC: 0.64***). In summer, reduced snow depth increased soil moisture (PC: -0.17*), thereby enhancing summer NPP (PC: 0.34***). Meanwhile, the delayed SOS reduced soil moisture deficit (PC: 0.11), further boosting summer NPP.

Overall, the “total effect” of snow depth on spring NPP via both the “SM effect” and the “GS effect” was positive in all ecological zones (**Figure 4G**). That is, regions that have experienced an increase in snow depth over the past four decades have also witnessed a concurrent increase in spring NPP, and areas with decreased snow depth have exhibited a decreased spring NPP. The “total effect” of snow depth on summer NPP was positive in the central-eastern TP (HIB1, HIC1, HIIAB1, and HIIC1) (**Figure 4H**), which means that an increased snow depth augmented the summer NPP in these areas. In contrast, the “total effect” was negative in the southwestern TP (such as HIC2 and HIIC2), meaning that a reduced snow depth augmented summer NPP.

### 3.3 The regulatory role of elevation on various ecological processes

We extracted the path coefficient values for each pixel, analyzed the variation pattern of these path coefficients with elevation, and defined the elevation intervals used in the analysis at intervals of 500 meters.

In spring, the path coefficients related to the GS effect (SDmax-SOS, SOS-NPP) exhibited significant elevational trends (**Figures 7A–C**). Specifically, the cascade effect of decreased snow depth - delayed SOS - decreased spring NPP intensified significantly as elevation increased. This indicates that elevation amplifies the cascading impacts of environmental factors (e.g., snow depth) on phenology and carbon sink. Moreover, the relationship between SOS and soil moisture also showed a significant elevational trend: it was negative below 4500 m but turned positive above this threshold (**Figure 7D**). For instance, in low-elevation regions such as the eastern TP (e.g., HIIC1 and HIB1), an earlier SOS led to increased soil moisture (PC: -0.43***/-0.39***), whereas in high-elevation regions such as the western TP (e.g., HIC2 and HIIC2), a delayed SOS reduced soil moisture consumption (PC: 0.35*/0.38**).

**Figure 7:**
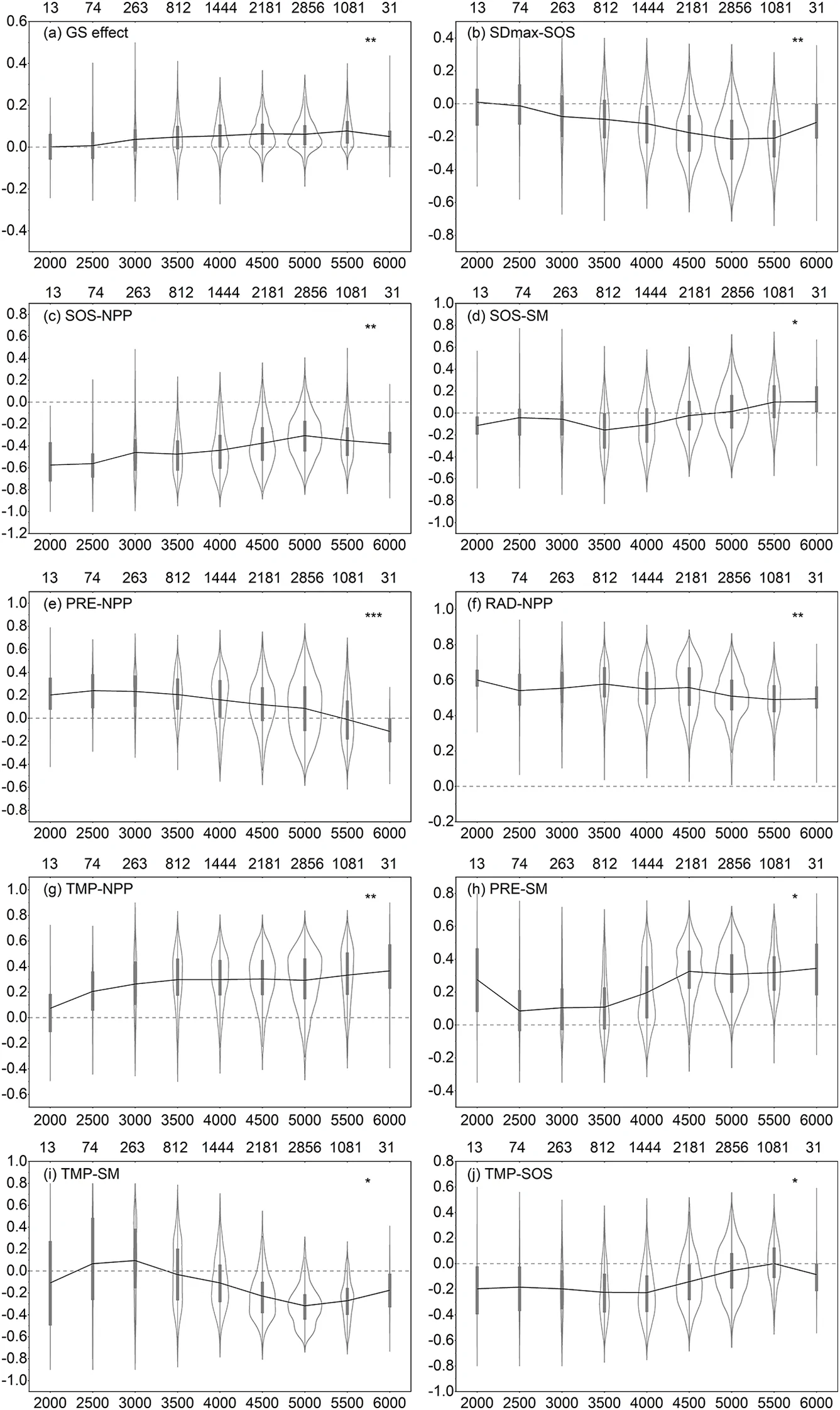
he variation trends of path coefficients regulating NPP through the growing season effect (GS effect) and the soil moisture effect (SM effect) with altitude in spring (only path coefficients with significant (P<0.05) variation trends are displayed). **(a)** GS effect; **(b)** SDmax–SOS; **(c)** SOS–NPP; **(d)** SOS–SM; **(e)** PRE–NPP; **(f)** RAD–NPP; **(g)** TMP–NPP; **(h)** PRE–SM; **(i)** TMP–SM; and **(j)** TMP–SOS. SDmax, maximum snow depth; SOS, start of the growing season; SM, soil moisture; PRE, precipitation; RAD, radiation; TMP, temperature. *indicates significance at the 0.05 level, **indicates significance at the 0.01 level, and ***indicates significance at the 0.001 level. Numbers above each panel indicate the number of pixels in the corresponding elevation bins.

In summer, the path coefficients associated with the SM effect (e.g., SDmax-SM) exhibited a significant elevational trend, leading to notable elevation-dependent patterns in both the SM effect and the total effect (**Figures 8A–C**). Specifically, the path coefficient between snow depth and summer soil moisture (SDmax-SM) showed a distinct elevational transition: below 4500 m, snow depth correlated positively with soil moisture, with the strength of this positive correlation decreasing with elevation. Above 4500m, the relationship turned negative, and the magnitude of the negative correlation increased with elevation. This pattern likely reflects a shift in the dominant hydrological control along the elevation gradient. At lower elevations (<4500 m), snowmelt serves as a key water source for soil moisture. Above 4500 m, where warming has been most pronounced and snow depth has declined substantially, the timing of soil thaw becomes more critical. Reduced snow depth may be associated with earlier thaw, potentially enhancing the soil’s capacity to retain subsequent summer rainfall. These changes promote earlier soil thawing and increase direct infiltration of summer precipitation, ultimately creating the seemingly counterintuitive pattern in which reduced snow depth at high elevations corresponds to increased soil moisture—as observed, for example, in the HIIC2 region of the southwestern TP.

**Figure 8:**
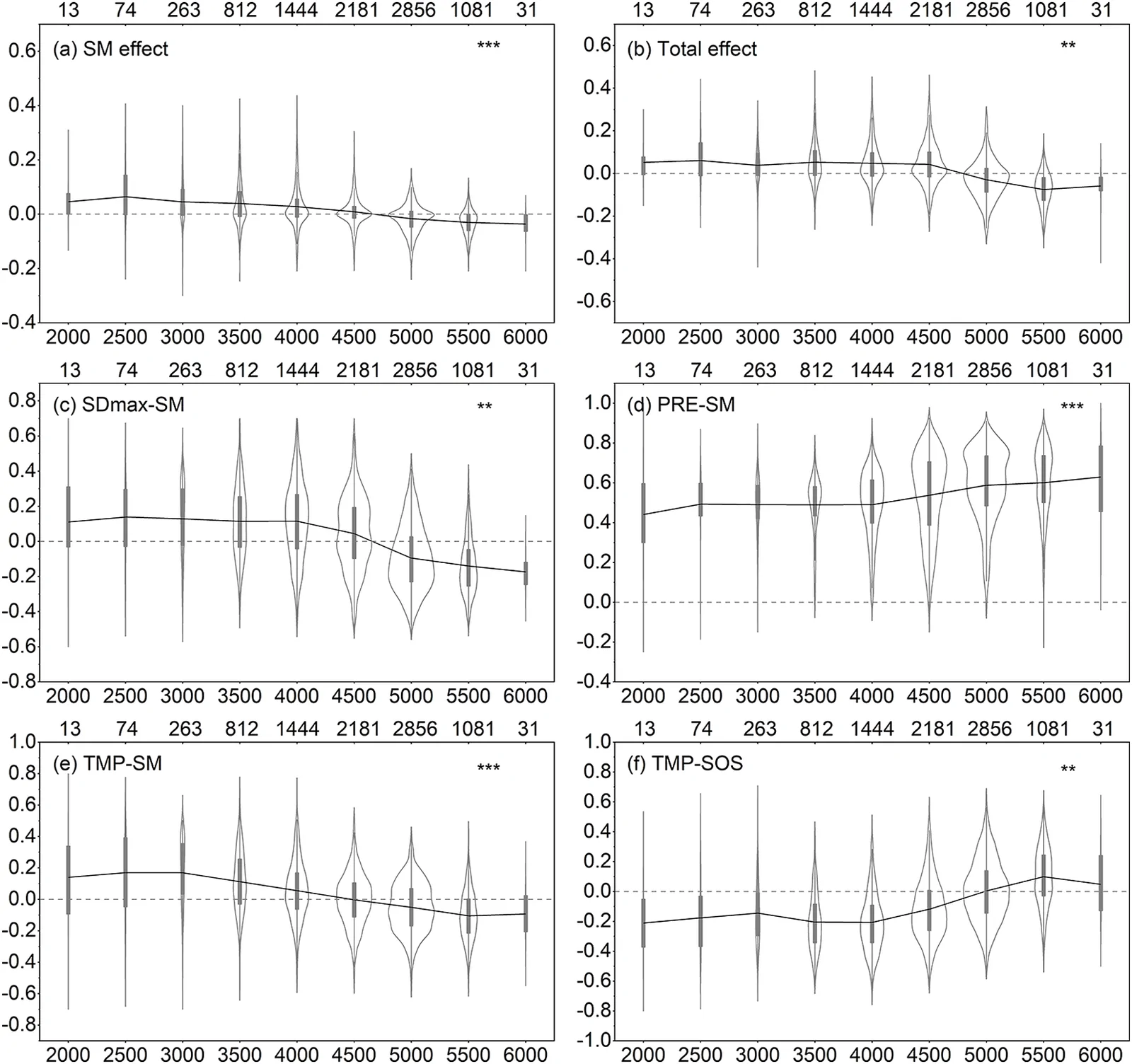
The variation trends of path coefficients regulating NPP through the growing season effect (GS effect) and the soil moisture effect (SM effect) with altitude in summer (only path coefficients with significant (P<0.05) variation trends are displayed). **(a)** SM effect; **(b)** Total effect; **(c)** SDmax–SM; **(d)** PRE–SM; **(e)** TMP–SM; and **(f)** TMP–SOS. SDmax, maximum snow depth; SOS, start of the growing season; SM, soil moisture; PRE, precipitation; TMP, temperature. *indicates significance at the 0.05 level, **indicates significance at the 0.01 level, and ***indicates significance at the 0.001 level. Numbers above each panel indicate the number of pixels in the corresponding elevation bins.

The path coefficients between NPP and spring precipitation, solar radiation, and temperature all exhibited significant elevational trends (**Figures 7E–G**). Specifically, the positive correlation between NPP and temperature increased with elevation, whereas the positive correlations between NPP and both precipitation and solar radiation decreased with elevation. This suggests that temperature becomes the primary limiting factor for NPP at higher elevations, while the limiting effects of precipitation and solar radiation weaken. At very high elevations, increased precipitation may even suppress NPP accumulation. In contrast, these path coefficients showed no significant elevational trends in summer, likely due to generally favorable climatic conditions that may have masked the regulating effects of elevation.

Elevation also modulated relationships between soil moisture and climatic factors (temperature and precipitation) during both spring and summer (**Figures 7H, I**, **8D, E**). The positive correlation between precipitation and soil moisture strengthened as elevation increased. In contrast, the relationship between temperature and soil moisture exhibited an elevation-dependent shift: below 4500 m, the two variables were positively correlated, but the strength of this positive correlation weakened with elevation. Above 4500 m, the correlation became negative, and the magnitude of this negative relationship increased with elevation (**Figure 8E**).

The correlation between SOS and summer temperature exhibited a significant elevational trend (**Figure 8F**). Below 4500 m, SOS correlated negatively with temperature, and the strength of this negative correlation weakened with increasing elevation. Above 4500 m, the correlation turned positive, and the strength of the positive correlation increased with elevation. This indicates that, with warming tending to advance SOS, vegetation at lower elevations is highly sensitive to temperature change. In contrast, at higher elevations, rising temperatures may delay SOS. The elevation around 4500 m may represent a transition zone where the coupling between hydrothermal conditions and phenological responses exhibits elevation-dependent shifts.

## 4 Discussion

### 4.1 Regional and seasonal variation in NPP regulated by snow depth via the growing season effect

Previous studies have reported that the growing season effect (GS effect) is predominantly negative in Central Europe, whereby increased snow depth (SDmax) delays snowmelt onset (SCM), subsequently postponing the start of the growing season (SOS), shortening its duration (LOS), and ultimately reducing early-season net primary productivity (NPP) (Wang et al., 2018a). In contrast, studies conducted in temperate regions of China suggest that the GS effect varies across ecosystems: in arid grasslands, increased snow depth tends to advance the SOS, thereby enhancing early carbon uptake, while in humid grasslands, increased snow depth delays SOS and suppresses early-season carbon uptake (Chen et al., 2019). On the Tibetan Plateau (TP), previous findings have revealed pronounced spatial heterogeneity in snow and phenology interactions. In the eastern TP (semi-humid area), increased snow depth and delayed snowmelt tend to advance SOS, whereas decreased snow depth and earlier snowmelt paradoxically lead to a delayed SOS in the western TP (semi- arid area) (**Supplementary Figures S4c, d**) (Huang et al., 2017; Ma et al., 2025).

Our study further substantiates these regional differences and highlights a seasonal divergence in the role of snow depth in regulating early-season NPP via its impact on SOS. Specifically, the GS effect was found to be positive across all ecozones during spring (**Figure 4G**). In the summer, it remained positive in semi-humid regions of the eastern and southern TP (e.g., HIB1, HIIAB1, HIIC1) but shifted to a negative effect in the semi-arid central and western regions (e.g., HIC1, HIC2, HIIC2) (**Figure 4H**). This spatial divergence may be attributed to variation in snow depth. In the eastern TP, increased precipitation during the snow accumulation season increases snow depth and delays the snowmelt period (Ma et al., 2023). This extended snowmelt process helps to maintain soil moisture, promoting a synergistic effect between water and heat that accelerates the SOS (Choler et al., 2025; Ma et al., 2025), thereby increasing spring and summer NPP.

In the semi-arid regions of the central and western TP, however, the rise in temperature during the snow accumulation season is associated with reduced snow depth and an earlier snowmelt period (Ma et al., 2023), which may contribute to lower spring soil moisture availability and a delayed SOS (Ma et al., 2025). This, in turn, delays the peak of the growing season (Huang et al., 2023), ultimately resulting in a lowered spring NPP and a heightened summer NPP. These findings are consistent with previous studies. For example, Zheng et al. (2020) demonstrated that an advanced SOS directly promotes spring NPP, whereas its lagged effects reduce summer productivity gains and suppress autumn productivity. Similarly, Liu et al. (2025a) indicated that seasonal productivity is constrained by the advancement of SOS and the peak of the growing season, which may limit carbon absorption later in the growing season. Furthermore, existing evidence suggests that climate-driven SOS advancement intensifies spring transpiration, reduces spring soil moisture, and may continue to trigger feedback effects on summer soil moisture through soil–atmosphere interactions (Lian et al., 2020). Especially in arid areas, early SOS significantly aggravates soil moisture deficiency (Liu et al., 2025a). Our results support this mechanism. In the semi-arid western TP (e.g., HIC2), a significant positive correlation was observed between SOS and soil moisture, and it persisted into summer (**Figures 6E, F**). In contrast, the semi-humid regions of the eastern TP (e.g., HIB1, HIIC1) exhibited a significant negative correlation between SOS and soil moisture (**Figure 6E**). This phenomenon may result from the region’s relatively sufficient supply of precipitation and snowmelt, whereby earlier greening enhances the hydrological cycle and consequently contributes to the maintenance or even increase of soil moisture (Zeng et al., 2018).

### 4.2 Regional and seasonal variation in NPP regulated by snow depth via the soil moisture effect

Soil moisture (SM) plays a paramount role in maintaining plant growth (Luo et al., 2021). Previous studies have shown that snow cover primarily exerts a positive effect on vegetation growth by enhancing soil moisture in regions such as central and western North America and the Greater Himalayas (Wang et al., 2018a). This mechanism is relatively intuitive: increased snow depth or the snow water equivalent typically contributes to heightened soil moisture content, thereby providing sufficient water for plant growth and promoting early accumulation of NPP (Wang et al., 2018a). However, on the TP, the response of vegetation to snow depth exhibits significant spatial heterogeneity. Existing research indicates that vegetation growth in the eastern TP is significantly positively correlated with snow water equivalent, while a significant negative correlation has been observed in the western TP (Chen et al., 2019). This study further confirms this pattern and reveals the spatiotemporal variability of the influence of snow depth on vegetation growth via soil moisture. Specifically, in the semi-humid zones of the central and eastern TP (e.g., HIC1, HIIAB1, HIIC1), snow depth influences spring and summer NPP through a consistently positive soil moisture effect (SM effect). In contrast, in the semi-arid zones of the southwestern TP (e.g., HIIC2), this effect exhibits seasonal variation, where the SM effect on NPP is positive in spring and turns negative during summer (**Figures 4G, H**). This discrepancy may be attributed to regional climatic characteristics. The HIIC2 region experiences a relatively shallow snow depth (**Figure 1D**), and it has significantly decreased further over the past four decades (**Figure 5A**), which was associated with an earlier onset of the snowmelt period (**Supplementary Figures S4d**, **S5b**). Consequently, the limited meltwater primarily benefits spring soil moisture (**Supplementary Figure S4a**), whereas summer soil moisture is mainly influenced by precipitation (PC: 0.79***). Although increased precipitation is generally considered to promote NPP growth, this study found a negative correlation between spring precipitation and NPP in approximately 32% of the areas in the central and eastern TP (**Supplementary Figure S2g**), those which are primarily classified as humid and semi-humid zones. This finding aligns with previous studies, including that of Schuur (2003), who suggested that precipitation may be negatively associated with NPP in moist ecosystems. Further, excess water may suppress plant uptake and utilization of other critical resources, such as soil nitrogen (Schuur and Matson, 2001; Huston, 2012).

Overall, the regulation of vegetation NPP by snow depth on the TP exhibits pronounced seasonal divergence. In spring, the “growing-season effect” predominates, where increased/decreased snow depth is associated with an advanced/delayed SOS, which may subsequently affect NPP. In contrast, summer NPP is dominated by the soil moisture effect (SM effect), whereby increased/decreased snow depth is associated with increased/decreased soil moisture, which may in turn contribute to higher/lower NPP. This seasonal difference is primarily related to the distinct roles of snow in constraining plant growth across seasons. In spring, snow depth directly determines the timing of snowmelt, which influences the onset of the growing season and controlling the accumulation of early-season productivity (Wang et al., 2018a; Wang et al., 2024; Ma et al., 2025). However, as summer arrives, temperature is no longer the primary limiting factor, and water availability becomes the critical constraint for vegetation growth and photosynthesis (Xu et al., 2023; Xie et al., 2024). Snow depth also has a significant lagged effect on soil moisture. Approximately 33% of the pixels showed a stronger correlation between snow depth and summer soil moisture compared to spring soil moisture. These areas, primarily in the western TP, exhibit negative correlations. In contrast, the remaining 67%, mainly in central and eastern TP, shows positive correlations (**Supplementary Figure S6**). Over the past four decades, snow depth has increased in the eastern TP (**Figure 5A**), positively influencing soil moisture, with effects that can persist into summer (**Supplementary Figure S6**). In contrast, despite a decrease in snow depth in the western TP (**Figure 5A**), both spring and summer soil moisture have increased (**Figures 5K, L**). This trend is largely attributable to the concurrent increase in precipitation (**Figures 5I, J**), which alleviates water stress in the arid conditions of the TP, thereby promoting the accumulation of summer vegetation NPP. These findings suggest that future climate warming, along with changes in snow depth, may have significant impacts on the regional carbon cycle.

### 4.3 Elevation modulates the impact of snow depth on vegetation growth

The influence of different ecological processes on vegetation growth is largely regulated by elevation. This study found that the path coefficients related to the GS effect exhibited significant altitudinal trends in the spring. For example, the reduction in snow depth led to a delay in the SOS, which in turn triggered cascading effects that lowered NPP. This effect significantly intensified as elevation increased. This could be because spring marks the initiation of plant growth, and changes in SOS related to snow depth and temperature, both of which exhibit significant elevation-based spatial variation. Specifically, the rate of snow depth reduction and the rate of temperature increase both rise with elevation (Ma et al., 2023). How SOS responds to these climate factors also shows a distinct elevation trend, and this in turn drives changes in NPP across different elevation gradients through SOS variation. Entering the summer, as temperatures continue to rise, snow depth primarily regulates vegetation NPP indirectly by affecting soil moisture. At this time, path coefficients related to soil moisture (e.g., SDmax-SM) exhibit a significant elevation trend. Notably, we observed an elevation-dependent shift in the relationships among several ecological variables around 4500 m elevation in this study. For instance, snow depth and summer soil moisture correlated positively below 4500m, but they became negatively correlated above 4500m. This may be because lower elevations can more effectively absorb snow meltwater, whereas frozen soils limit water infiltration at higher elevations, thereby resulting in lower soil moisture despite greater snow accumulation (Wang et al., 2023). Furthermore, the reduction in available water at high elevations further limits and counteracts vegetation greening (Liu et al., 2019). The correlation between temperature and soil moisture also shifts from positive to negative at this elevation, while the correlation between SOS and temperature shifts from negative to positive. These findings suggest that 4500m may represent an important boundary between the water-heat coupling mechanism and vegetation phenological responses, ultimately reflecting a dramatic shift in water-heat combinations, snow dynamics, and vegetation adaptation. This shift may reshape ecosystem structure, water resource distribution, and climate feedback processes (Wieder et al., 2022). Future climate change may exacerbate this differentiation (Gui et al., 2024), thus highlighting the need for long-term monitoring and modeling to predict the cascading impacts on hydrology, ecology, and carbon cycle.

### 4.4 Limitations and future perspectives

A passive microwave snow depth dataset provides reliable daily snow depth data with a long time series (1982-2020) for monitoring snow cover changes over the TP. However, the relatively coarse spatial resolution (25 km) of passive microwave remote sensing may limit the accuracy of the retrieved snow depth. For instance, low ground temperatures can lead to overestimation of snow depth in the northwest and southeast TP, while areas with shallow snow depth and cold desert in the midwest TP may suffer from underestimation (Dai et al., 2017). Optical satellite remote sensing products, such as MODIS and Landsat, have great potential in monitoring snow changes in complex terrain areas (Bayle et al., 2025). However, the similarity in reflectance spectra between snow cover and clouds makes snow monitoring via optical remote sensing highly susceptible to weather conditions (Dariane et al., 2017; Huang et al., 2018). Additionally, the lack of snow depth detection methods also limits the application of optical remote sensing snow products (Dai et al., 2017). While satellites like ICESat-2, which uses high-precision elevation change detection to monitor snow depth variations, currently provide valuable data (Deschamps-Berger et al., 2023), their spatial continuity is insufficient for large-scale studies in regions such as the TP, and their temporal frequency does not meet the needs of this study. However, in the future, those high-resolution satellite remote sensing products could be combined with machine learning downscaling techniques to spatially downscale passive microwave snow depth data, thereby generating high-temporal and high-spatial resolution snow depth datasets for more refined studies.

In this study, we identified two potential pathways linking snow depth to NPP. However, these pathways should be interpreted as potential mechanisms rather than confirmed causal relationships. Furthermore, the mechanisms through which snow depth influences NPP are considerably more complex than those identified in this study. For instance, the insulating effect of snow can elevate soil temperature, enhance microbial activity, and promote the decomposition of soil organic matter and nitrogen mineralization in cold regions such as the Arctic (Schimel et al., 2004; Brooks et al., 2011; Liu et al., 2023; Cui et al., 2024), thus increasing the availability of nutrients for plants. As a result, an increase in snow depth may enhance nutrient availability and, consequently, ecosystem productivity (Choler, 2015). This may help explain why thicker snow depth and delayed snowmelt advance SOS on the TP, thereby boosting spring NPP. Furthermore, permafrost, as an important underlying surface component of the Tibetan Plateau, may influence the effects of snow on vegetation productivity by regulating soil hydrothermal processes, active-layer freeze-thaw dynamics, and water availability (Zhao et al., 2021; Fu et al., 2026). The shift in the relationships among multiple variables observed around 4500 m in this study may be partly associated with variations in permafrost thermal conditions and active-layer dynamics. At lower elevations, greater snow depth is generally accompanied by increased meltwater input, which may improve spring soil moisture conditions and influence vegetation green-up timing. However, in high-elevation regions with widespread permafrost, the effects of snow on vegetation are more complex. These effects may be jointly regulated by snow insulation, active-layer thickness, and freeze-thaw processes (Zhao et al., 2021). Greater snow accumulation may reduce winter heat loss from the ground surface and alter soil thermal conditions, potentially affecting spring thawing processes and active-layer development. Meanwhile, permafrost conditions may influence water migration processes by limiting deep soil infiltration, resulting in soil moisture dynamics being largely controlled within the active layer (Wang et al., 2009; Lv et al., 2022). Therefore, the response of soil moisture to snow variation may differ across contrasting permafrost environments. In regions experiencing permafrost degradation or transitional states, snow insulation may alter soil freeze-thaw dynamics and potentially reduce the availability of water during the growing-season, thereby weakening the positive association between increased snow cover and NPP (Li et al., 2024; Lv et al., 2022). In contrast, under relatively stable permafrost conditions, moderate snow cover may maintain higher soil temperatures, promote active-layer thawing, and enhance water availability, potentially creating more favorable conditions for vegetation growth (Wang et al., 2009). These interpretations should be considered as potential explanations for the observed elevation-dependent patterns, rather than direct evidence of permafrost-driven mechanisms, because permafrost conditions were not explicitly quantified in this study.

In addition, the GLEAM root-zone soil moisture product (10–100 cm) used in this study has certain limitations in permafrost regions. The model does not fully represent the freeze-thaw processes and water phase transitions characteristic of permafrost environments, which may lead to insufficient representation of the mid-active-layer “dry layer” phenomenon and its constraints on root-accessible water availability (Wang et al., 2009; Yang et al., 2022). Therefore, future applications of GLEAM products in permafrost regions should integrate process-based models and high-resolution observations to improve the representation of water-heat-vegetation interactions. Moreover, incorporating permafrost thermal processes, active-layer dynamics, and vegetation-soil hydrothermal feedback into ecosystem models will be essential for improving predictions of carbon cycle dynamics in alpine ecosystems on the Tibetan Plateau.

Finally, it should be noted that soil moisture stress is explicitly incorporated into CASA-based NPP estimation. Therefore, the soil moisture pathway identified in this study may be partly influenced by the model structure. This pathway should be interpreted as the combined outcome of snow-induced changes in water availability and the model representation of moisture limitation, rather than as an independent causal effect of soil moisture on NPP. Particularly in permafrost regions, the regulation of soil water availability by freeze-thaw processes and active-layer dynamics requires further evaluation using process-based models and independent observations.

It should be noted that the precipitation data used in this study are total precipitation, without further distinguishing between snowfall and rainfall. Different precipitation phases have significantly different effects on the efficiency of soil moisture replenishment and the amount of water available to vegetation, which may to some extent affect the accurate quantification of the soil moisture pathway. Furthermore, the model does not account for the potential regulatory effect of wind speed on soil moisture—wind speed can indirectly influence soil moisture conditions through processes such as altering surface evapotranspiration, wind-driven snow redistribution, and sublimation losses (Fang and Pomeroy, 2020; Marsh et al., 2023). Overall, although temperature, precipitation, and solar radiation were selected as the main climatic variables in this study, these choices still have certain limitations, particularly in terms of data resolution and the diversity of climatic factors. Future data collection and model improvements could consider incorporating more detailed precipitation type differentiation and wind speed factors to enhance the accuracy and comprehensiveness of the research.

It is also important to note that the mechanisms through which snow cover influences vegetation growth vary by vegetation type. Different biomes require distinct environmental conditions to break dormancy and resume growth in spring (Xu et al., 2022; Xiao et al., 2023). For instance, trees typically require a certain amount of cold accumulation to complete their dormancy phase (Harrington and Gould, 2015; Rodríguez et al., 2019), while herbaceous plants are more reliant on early-spring warming signals (Alecrim et al., 2023). Consequently, the insulating effect of snow may have divergent impacts on different plant communities (Chen et al., 2019). In summary, snow cover can influence vegetation growth not only by altering soil temperature, moisture, and phenological conditions but also through its effects on nutrient availability and freeze-thaw frequency. These processes are modulated by a range of interacting factors, including regional climate, vegetation type, and soil characteristics. Current structural equation models have yet to fully capture these complex mechanisms. Future research should integrate multi-source remote sensing data, ground-based observations, and ecological process models to further explore the multi-pathway impacts of snow changes on NPP across different ecosystems.

## 5 Conclusions

Based on multi-source remote sensing and long-term field observation data, this study employed a structural equation model to elucidate the climate–biological regulatory mechanisms through which snow depth influences spring and summer vegetation net primary productivity (NPP) on the Tibetan Plateau (TP). The results reveal distinct seasonal and regional differences in the impact of snow depth on vegetation NPP. In the semi-humid regions of the central and eastern TP, increased snow depth and delayed snowmelt over the past four decades have enhanced spring and summer soil moisture, thereby advanced the start of the growing season (SOS) and promoting higher NPP during both seasons. Conversely, in the semi-arid southwestern TP, reduced snow depth and earlier snowmelt have extended pre-season bare ground exposure, which was associated with spring soil moisture deficits, delayed SOS, and reduced spring NPP, but also increased summer NPP. Moreover, the influence of ecological processes on vegetation growth is profoundly regulated by elevation. An elevation of approximately 4500 m may represent a transition zone where the relationships among several key ecological variables exhibit elevation-dependent shifts, reflecting potential changes in snow dynamics, hydrothermal conditions, and vegetation responses along the elevation gradient. Ongoing climate change is expected to intensify these spatial discontinuities, with potential consequences for ecosystem structure, water resource allocation, and climate feedback mechanisms. These pronounced regional differences and vertical heterogeneity indicate that the impact of snow depth on vegetation productivity is not consistent across the elevation gradient. Our results show that the strength and even the direction of several key pathways vary significantly with elevation, with a reversal observed around 4500 m. This reflects the nonlinear changes in climate effects and vegetation adaptation strategies along the altitudinal gradient. Our findings provide new insight into the adaptive strategies of alpine vegetation under changing climatic conditions and enrich the theoretical framework for understanding vegetation dynamics on the Tibetan Plateau in the context of global climate change.

## Data Availability

The original contributions presented in the study are included in the article/**Supplementary Material**. Further inquiries can be directed to the corresponding authors.

## References

[B1] Alecrim E. F. Sargent R. D. Forrest J. R. K. (2023). Higher‐latitude spring‐flowering herbs advance their phenology more than trees with warming temperatures. J. Ecol. 111, 156–169. doi: 10.1111/1365-2745.1402340046247

[B2] Bai L. Tian L. Ren Z. Song X. Yu K. Meng L. . (2024). Climate warming advances plant reproductive phenology in China’s northern grasslands. J. Plant Ecol. 17, rtae080. doi: 10.1093/jpe/rtae080

[B3] Bayle A. Gascoin S. Corona C. Stoffel M. Choler P. (2025). Snow melt-out date (SMOD) change spanning four decades in European temperate mountains at 30 m from landsat time series. Sci. Data 12, 706. doi: 10.1038/s41597-025-05044-2 40301341 PMC12041387

[B4] Bibi S. Wang L. Li X. Zhou J. Chen D. Yao T. (2018). Climatic and associated cryospheric, biospheric, and hydrological changes on the Tibetan plateau: A review. Int. J. Climatol. 38, e1–e17. doi: 10.1002/joc.541142587375

[B5] Brooks P. D. Grogan P. Templer P. H. Groffman P. Öquist M. G. Schimel J. (2011). Carbon and nitrogen cycling in snow-covered environments: Carbon and nitrogen cycling in snow-covered environments. Geogr. Compass. 5, 682–699. doi: 10.1111/j.1749-8198.2011.00420.x40046247

[B6] Buermann W. Forkel M. O’Sullivan M. Sitch S. Friedlingstein P. Haverd V. . (2018). Widespread seasonal compensation effects of spring warming on northern plant productivity. Nature 562, 110–114. doi: 10.1038/s41586-018-0555-7 30283105

[B7] Che T. Dai L. Y. (2015). Long-term series of daily snow depth dataset in China (1979-2021). National Tibetan Plateau Data Center. doi: 10.11888/Geogra.tpdc.270194

[B8] Che T. Dai L. Zheng X. Li X. Zhao K. (2016). Estimation of snow depth from passive microwave brightness temperature data in forest regions of northeast China. Remote Sens. Environ. 183, 334–349. doi: 10.1016/j.rse.2016.06.00542574925

[B9] Chen S. Huang Y. Wang G. (2019). Response of vegetation carbon uptake to snow-induced phenological and physiological changes across temperate China. Sci. Total. Environ. 692, 188–200. doi: 10.1016/j.scitotenv.2019.07.222 31349162

[B10] Chen X. Long D. Hong Y. Hao X. Hou A. (2018). Climatology of snow phenology over the Tibetan plateau for the period 2001–2014 using multisource data. Int. J. Climatol. 38, 2718–2729. doi: 10.1002/joc.545542587375

[B11] Choler P. (2015). Growth response of temperate mountain grasslands to inter-annual variations in snow cover duration. Biogeosciences 12, 3885–3897. doi: 10.5194/bg-12-3885-2015

[B12] Choler P. Bayle A. Fort N. Gascoin S. (2025). Waning snowfields have transformed into hotspots of greening within the alpine zone. Nat. Clim. Change 15, 80–85. doi: 10.1038/s41558-024-02177-x37880705

[B13] Cui K. Wang Y. Zhang X. Zhang X. Zhang X. Li Y. . (2024). Archaeal communities change responding to anthropogenic and natural treatments of freeze-thawed soils. Environ. Res. 255, 119150. doi: 10.1016/j.envres.2024.119150 38763282

[B14] Dai L. Che T. Ding Y. Hao X. (2017). Evaluation of snow cover and snow depth on the Qinghai–Tibetan plateau derived from passive microwave remote sensing. Cryosphere 11, 1933–1948. doi: 10.5194/tc-11-1933-2017

[B15] Dai L. Che T. Wang J. Zhang P. (2012). Snow depth and snow water equivalent estimation from AMSR-E data based on a priori snow characteristics in Xinjiang, China. Remote Sens. Environ. 127, 14–29. doi: 10.1016/j.rse.2011.08.02942574925

[B16] Dang C. Shao Z. Huang X. Zhuang Q. Cheng G. Qian J. (2023). Climate warming-induced phenology changes dominate vegetation productivity in northern hemisphere ecosystems. Ecol. Indic. 151, 110326. doi: 10.1016/j.ecolind.2023.11032642574925

[B17] Dariane A. B. Khoramian A. Santi E. (2017). Investigating spatiotemporal snow cover variability via cloud-free MODIS snow cover product in central Alborz region. Remote Sens. Environ. 202, 152–165. doi: 10.1016/j.rse.2017.05.04242574925

[B18] Deschamps-Berger C. Gascoin S. Shean D. Besso H. Guiot A. López-Moreno J. I. (2023). Evaluation of snow depth retrievals from ICESat-2 using airborne laser-scanning data. Cryosphere 17, 2779–2792. doi: 10.5194/tc-17-2779-2023

[B19] Fang X. Pomeroy J. W. (2020). Diagnosis of future changes in hydrology for a Canadian Rockies headwater basin. Hydrol. Earth Syst. Sci. 24, 2731–2754. doi: 10.5194/hess-24-2731-2020

[B20] Fu Z. Wang L. Jiang G. Men X. Du W. Yang Y. . (2026). Decadal-scale thermal memory of permafrost and climatic and topographic modulation on the Tibetan Plateau. NPJ Clim. Atmos. Sci. 9, 100. doi: 10.1038/s41612-026-01368-x37880705

[B21] Grace J. B. (2006). Structural Equation Modeling and Natural Systems. (Cambridge: Cambridge University Press).

[B22] Gu H. Qiao Y. Xi Z. Rossi S. Smith N. G. Liu J. . (2022). Warming-induced increase in carbon uptake is linked to earlier spring phenology in temperate and boreal forests. Nat. Commun. 13, 3698. doi: 10.1038/s41467-022-31496-w 35760820 PMC9237039

[B23] Gui K. Zhou T. Zhang W. Zhang X. (2024). Land-atmosphere coupling amplified the record-breaking heatwave at altitudes above 5000 meters on the Tibetan plateau in july 2022. Weather. Clim. Extrem. 45, 100717. doi: 10.1016/j.wace.2024.10071742574925

[B24] Hair J. F. Hult G. T. M. Ringle C. M. Sarstedt M. Danks N. P. Ray S. (2021). “ An introduction to structural equation modeling,” in Partial Least Squares Structural Equation Modeling (Pls-Sem) Using R: A Workbook. Eds. Hair J. F. Hult G. T. M. Ringle C. M. Sarstedt M. Danks N. P. Ray S. ( Springer International Publishing, Cham), 1–29. doi: 10.1007/978-3-030-80519-7_1

[B25] Harrington C. A. Gould P. J. (2015). Tradeoffs between chilling and forcing in satisfying dormancy requirements for pacific northwest tree species. Front. Plant Sci. 6, 1. doi: 10.3389/fpls.2015.00120 25784922 PMC4347443

[B26] Huang X. Deng J. Wang W. Feng Q. Liang T. (2017). Impact of climate and elevation on snow cover using integrated remote sensing snow products in Tibetan plateau. Remote Sens. Environ. 190, 274–288. doi: 10.1016/j.rse.2016.12.02842574925

[B27] Huang Y. Liu H. Yu B. Wu J. Kang E. L. Xu M. . (2018). Improving MODIS snow products with a HMRF-based spatio-temporal modeling technique in the upper rio grande basin. Remote Sens. Environ. 204, 568–582. doi: 10.1016/j.rse.2017.10.00142574925

[B28] Huang Z. Zhou L. Chi Y. (2023). Spring phenology rather than climate dominates the trends in peak of growing season in the northern hemisphere. Glob. Change Biol. 29, 4543–4555. doi: 10.1111/gcb.16758 37198735

[B29] Huston M. A. (2012). Precipitation, soils, NPP, and biodiversity: Resurrection of albrecht’s curve. Ecol. Monogr. 82, 277–316. doi: 10.1890/11-1927.1

[B30] Immerzeel W. W. van Beek L. P. H. Bierkens M. F. P. (2010). Climate change will affect the Asian water towers. Science 328 (5984), 1382–1385. doi: 10.1126/science.1183188 20538947

[B31] Irannezhad M. Ronkanen A.-K. Malekian A. (2022). Editorial: Climate impacts on snowpack dynamics. Front. Earth Sci. 10, 970981. doi: 10.3389/feart.2022.970981

[B32] Jönsson P. Eklundh L. (2004). TIMESAT—a program for analyzing time-series of satellite sensor data. Comput. Geosci. 30, 833–845. doi: 10.1016/j.cageo.2004.05.00642574925

[B33] Kang S. C. Xu Y. W. You Q. L. Flügel W. Pepin N. Yao T. D. (2010). Review of climate and cryospheric change in the Tibetan Plateau. Environ. Res. Lett. 5 (1), 015101. doi: 10.1088/1748-9326/5/1/015101

[B34] Kendall M. G. (1948). Rank correlation methods. (Oxford, England: Griffin).

[B35] Li H. Xiao P. Feng X. Yang Y. Wang L. Zhang W. . (2017). Using land long-term data records to map land cover changes in China over 1981–2010. IEEE J. Sel. Top. Appl. Earth Obs. Remote Sens. 10, 1372–1389. doi: 10.1109/JSTARS.2016.264520325079929

[B36] Li W. Yan D. Weng B. Lai Y. Zhu L. Qin T. . (2024). Nonlinear effects of surface soil moisture changes on vegetation greenness over the Tibetan Plateau. Remote Sens. Environ. 302, 113971. doi: 10.1016/j.rse.2023.11397142574925

[B37] Li M.-W. Zhang J. Wu Z.-F. Fu Y.-S. (2025). Effect of growing season length on gross primary productivity increased in the Jinsha river watershed. J. Plant Ecol. 18, rtae108. doi: 10.1093/jpe/rtae108

[B38] Lian X. Peñuelas J. Ryu Y. Piao S. Keenan T. F. Fang J. . (2024). Diminishing carryover benefits of earlier spring vegetation growth. Nat. Ecol. Evol. 8, 218–228. doi: 10.1038/s41559-023-02272-w 38172284

[B39] Lian X. Piao S. Li L. Z. X. Li Y. Huntingford C. Ciais P. . (2020). Summer soil drying exacerbated by earlier spring greening of northern vegetation. Sci. Adv. 6, eaax0255. doi: 10.1126/sciadv.aax0255 31922002 PMC6941915

[B40] Liu L. Wang Y. Wang Z. Li D. Zhang Y. Qin D. . (2019). Elevation-dependent decline in vegetation greening rate driven by increasing dryness based on three satellite NDVI datasets on the Tibetan plateau. Ecol. Indic. 107, 105569. doi: 10.1016/j.ecolind.2019.10556942574925

[B41] Liu H. Xiao P. Zhang X. Wu Y. (2023). Increased snow cover enhances gross primary productivity in cold and dry regions of the Tibetan plateau. Ecosphere 14, e4656. doi: 10.1002/ecs2.465642587375

[B42] Liu X. Li Z. X. Li Z. J. Liu M. Xu B. Li H. . (2025a). Impacts of various soil moisture replenishment patterns on plant water use strategies in the third pole region. CATENA 254, 108942. doi: 10.1016/j.catena.2025.10894242574925

[B43] Liu Z. Fu Y. H. Chen D. Crowther T. W. Ding R. Hao F. . (2025b). Increased early-season productivity drives earlier peak of vegetation photosynthesis across the northern hemisphere. Commun. Earth Environ. 6, 157. doi: 10.1038/s43247-025-02104-837880705

[B44] Luo M. Meng F. Sa C. Duan Y. Bao Y. Liu T. . (2021). Response of vegetation phenology to soil moisture dynamics in the Mongolian plateau. CATENA 206, 105505. doi: 10.1016/j.catena.2021.10550542574925

[B45] Lv M. Wang Y. Gao Z. (2022). The change process of soil hydrological properties in the permafrost active layer of the Qinghai–Tibet plateau. CATENA 210, 105938. doi: 10.1016/j.catena.2021.10593842574925

[B46] Ma Q. Keyimu M. Li X. Wu S. Zeng F. Lin L. (2023). Climate and elevation control snow depth and snow phenology on the Tibetan plateau. J. Hydrol. 617, 128938. doi: 10.1016/j.jhydrol.2022.12893842574925

[B47] Ma Q. Liu J. Li X. Li Y. Zeng F. Guo X. . (2025). Snow depth and spring temperature dominate the spring phenological shifts and control growing season dynamics on the Tibetan Plateau. Agric. For. Meteorol. 363, 110435. doi: 10.1016/j.agrformet.2025.11043542574925

[B48] Mann H. B. (1945). Nonparametric tests against trend. 245–259.

[B49] Marchin R. M. McHugh I. Simpson R. R. Ingram L. J. Balas D. S. Evans B. J. . (2018). Productivity of an Australian mountain grassland is limited by temperature and dryness despite long growing seasons. Agric. For. Meteorol. 256–257, 116–124. doi: 10.1016/j.agrformet.2018.02.03042574925

[B50] Marsh C. B. Vionnet V. Pomeroy J. W. (2023). Windmapper: An efficient wind downscaling method for hydrological models. Water Resour. Res. 59, e2022WR032683. doi: 10.1029/2022WR032683

[B51] Martens B. Miralles D. G. Lievens H. van der Schalie R. de Jeu R. A. M. Férnandez-Prieto D. (2017). GLEAM v3: Satellite-based land evaporation and root-zone soil moisture. Geoscientific Model Development 10, 1903–1925. doi: 10.5194/gmd-2016-162

[B52] Nury A. H. Sharma A. Mehrotra R. Marshall L. Cordery I. (2022). Projected changes in the Tibetan plateau snowpack resulting from rising global temperatures. J. Geophys. Res. Atmos. 127, e2021JD036201. doi: 10.1029/2021JD03620129143083

[B53] Peng S. Z. (2019). 1-km monthly mean temperature dataset for China (1901-2021). A Big Earth Data Platform for Three Poles. doi: 10.11888/Meteoro.tpdc.270961

[B54] Piao S. Liu Q. Chen A. Janssens I. A. Fu Y. Dai J. . (2019). Plant phenology and global climate change: Current progresses and challenges. Glob. Change Biol. 25, 1922–1940. doi: 10.1111/gcb.14619 30884039

[B55] Ren Y. Qiu J. Zeng Z. Liu X. Sitch S. Pilegaard K. . (2024). Earlier spring greening in northern hemisphere terrestrial biomes enhanced net ecosystem productivity in summer. Commun. Earth Environ. 5, 122. doi: 10.1038/s43247-024-01270-537880705

[B56] Rodríguez A. Pérez-López D. Sánchez E. Centeno A. Gómara I. Dosio A. . (2019). Chilling accumulation in temperate fruit trees in Spain under climate change. Nat. Hazards Earth Syst. Sci. 19, 1087–1103. doi: 10.5194/nhess-2018-392

[B57] Schimel J. P. Bilbrough C. Welker J. M. (2004). Increased snow depth affects microbial activity and nitrogen mineralization in two Arctic tundra communities. Soil Biol. Biochem. 36, 217–227. doi: 10.1016/j.soilbio.2003.09.00842574925

[B58] Schuur E. A. G. (2003). Productivity and global climate revisited: The sensitivity of tropical forest growth to precipitation. Ecology 84, 1165–1170. doi: 10.1890/0012-9658(2003)084[1165:PAGCRT]2.0.CO;2

[B59] Schuur E. A. Matson P. A. (2001). Net primary productivity and nutrient cycling across a mesic to wet precipitation gradient in Hawaiian montane forest. Oecologia 128, 431–442. doi: 10.1007/s004420100671 24549913

[B60] Shipley B. (2016). Cause and Correlation in Biology: A User’s Guide to Path Analysis, Structural Equations and Causal Inference with R. (Cambridge: Cambridge University Press).

[B61] Sun Y. Feng Y. Wang Y. Zhao X. Yang Y. Tang Z. . (2021). Field‐based estimation of net primary productivity and its above‐ and belowground partitioning in global grasslands. J. Geophys. Res. Biogeosciences. 126, e2021JG006472. doi: 10.1029/2021JG00647229143083

[B62] Tang S. Wang T. Liu D. Yao T. Piao S. (2024). Impacts of land surface darkening on frozen ground and ecosystems over the Tibetan plateau. Sci. China Earth Sci. 67, 3213–3225. doi: 10.1007/s11430-023-1363-330311153

[B63] Tong C. L. Zhang W. J. Tang Y. Wang H. Q. (2005). Estimation of Daily Solar Radiation in China. Chinese Journal of Agrometeorology 26 (3), 165–169.

[B64] Wang G. Li S. Hu H. Li Y. (2009). Water regime shifts in the active soil layer of the Qinghai–Tibet plateau permafrost region, under different levels of vegetation. Geoderma 149, 280–289. doi: 10.1016/j.geoderma.2008.12.00842574925

[B65] Wang L. Li P. Peng Y. Ren P. Chen Y. Zhou X. . (2025). The widely increasing sensitivity of vegetation productivity to phenology in northern middle and high latitudes. Geophys. Res. Lett. 52, e2024GL113892. doi: 10.1029/2024GL11389229143083

[B66] Wang J. Ouyang W. Liu X. Wang L. (2023). Monitoring hydrological changes with satellite data: Spring thaw’s effect on soil moisture and groundwater in seasonal freezing-thawing zones. J. Hydrol. 626, 130365. doi: 10.1016/j.jhydrol.2023.13036542574925

[B67] Wang X. Wang T. Guo H. Liu D. Zhao Y. Zhang T. . (2018a). Disentangling the mechanisms behind winter snow impact on vegetation activity in northern ecosystems. Glob. Change Biol. 24, 1651–1662. doi: 10.1111/gcb.13930 28994227

[B68] Wang X. Wu C. Peng D. Gonsamo A. Liu Z. (2018b). Snow cover phenology affects alpine vegetation growth dynamics on the Tibetan plateau: Satellite observed evidence, impacts of different biomes, and climate drivers. Agric. For. Meteorol. 256–257, 61–74. doi: 10.1016/j.agrformet.2018.03.00442574925

[B69] Wang Y. Xiao P. Zhang X. Liu H. Wu Y. Sun L. (2024). Unraveling the effects of snow cover change on vegetation productivity: Insights from underlying surface types. Ecosphere 15, e4855. doi: 10.1002/ecs2.485542587375

[B70] Wang X. Xie H. (2009). New methods for studying the spatiotemporal variation of snow cover based on combination products of MODIS terra and aqua. J. Hydrol. 371, 192–200. doi: 10.1016/j.jhydrol.2009.03.02842574925

[B71] Wang J. Zhang M. Wang S. Ren Z. Che Y. Qiang F. . (2016). Decrease in snowfall/rainfall ratio in the Tibetan plateau from 1961 to 2013. J. Geogr. Sci. 26, 1277–1288. doi: 10.1007/s11442-016-1326-830311153

[B72] Wieder W. R. Kennedy D. Lehner F. Musselman K. N. Rodgers K. B. Rosenbloom N. . (2022). Pervasive alterations to snow-dominated ecosystem functions under climate change. Proc. Natl. Acad. Sci. 119, e2202393119. doi: 10.1073/pnas.2202393119 35858427 PMC9335325

[B73] Xiao J. Wang Z. Sun W. Li S. Han F. Huang S. . (2023). The relative effects of climate change and phenological change on net primary productivity vary with grassland types on the Tibetan plateau. Remote Sens. 15, 3733. doi: 10.3390/rs1515373330654563

[B74] Xie J. Yin G. Ma D. Chen R. Zhao W. Xie Q. . (2024). Climatic limitations on grassland photosynthesis over the Tibetan plateau shifted from temperature to water. Sci. Total. Environ. 906, 167663. doi: 10.1016/j.scitotenv.2023.167663 37813264

[B75] Xu C. Liu D. Wang X. Wang T. (2023). Shifting from a thermal-constrained to water-constrained ecosystem over the Tibetan plateau. Front. Plant Sci. 14, 1125288. doi: 10.3389/fpls.2023.1125288 37152137 PMC10154622

[B76] Xu J. Tang Y. Xu J. Shu S. Yu B. Wu J. . (2022). Impact of snow cover phenology on the vegetation green-up date on the Tibetan plateau. Remote Sens. 14, 3909. doi: 10.3390/rs1416390930654563

[B77] Yang T. Li Q. Ahmad S. Zhou H. Li L. (2019). Changes in snow phenology from 1979 to 2016 over the Tianshan mountains, central Asia. Remote Sens. 11, 499. doi: 10.3390/rs1105049930654563

[B78] Yang Y. Xiao P. Feng X. Li H. (2017). Accuracy assessment of seven global land cover datasets over China. ISPRS. J. Photogramm. Remote Sens. 125, 156–173. doi: 10.1016/j.isprsjprs.2017.01.01642574925

[B79] Yang S. Zeng J. Fan W. Cui Y. (2022). Evaluating root-zone soil moisture products from GLEAM, GLDAS, and ERA5 based on in situ observations and triple collocation method over the Tibetan Plateau. J. Hydrometeorol. 23, 1861–1878. doi: 10.1175/JHM-D-22-0016.1

[B80] Yao T. D. Thompson L. Yang W. Yu W. S. Gao Y. Guo X. J. . (2012). Different glacier status with atmospheric circulations in Tibetan Plateau and surroundings. Nat. Clim. Change 2 (9), 663–667. doi: 10.1038/nclimate1580

[B81] Yin H. Ma X. Liao X. Ye H. Yu W. Li Y. . (2024). Linking vegetation phenology to net ecosystem productivity: Climate change impacts in the northern hemisphere using satellite data. Remote Sens. 16, 4101. doi: 10.3390/rs1621410130654563

[B82] You Q. Wu T. Shen L. Pepin N. Zhang L. Jiang Z. . (2020). Review of snow cover variation over the Tibetan plateau and its influence on the broad climate system. Earth Sci. Rev. 201, 103043. doi: 10.1016/j.earscirev.2019.10304342574925

[B83] Zeng Z. Piao S. Li L. Z. X. Wang T. Ciais P. Lian X. . (2018). Impact of earth greening on the terrestrial water cycle. J. Clim. 31, 2633–2650. doi: 10.1175/JCLI-D-17-0236.1

[B84] Zhang Y. L. Li B. Y. Zheng D. (2014). Datasets of the boundary and area of the Tibetan Plateau. Acta. Geogr. Sin. 69, 65–68. doi: 10.11821/dlxb2014S012

[B85] Zhao L. Hu G. Wu X. Wu T. Li R. Pang Q. . (2021). Dynamics and characteristics of soil temperature and moisture of active layer in the central Tibetan plateau. Geoderma 400, 115083. doi: 10.1016/j.geoderma.2021.11508342574925

[B86] Zheng D. Yang Q. Wu S. (2008). Research on China’s Ecogeographical Regionalization System. (Beijing: The Commercial Press).

[B87] Zheng J. Xu X. Jia G. (2022). Effects of shifting spring phenology on growing season carbon uptake in high latitudes. J. Geophys. Res. Biogeosciences. 127, e2022JG006900. doi: 10.1029/2022JG00690029143083

[B88] Zheng Z. Zhu W. Zhang Y. (2020). Direct and lagged effects of spring phenology on net primary productivity in the alpine grasslands on the Tibetan plateau. Remote Sens. 12, 1223. doi: 10.3390/rs1207122330654563

[B89] Zhu W. Pan Y. He H. Yu D. Hu H. (2006). Simulation of maximum light use efficiency for some typical vegetation types in China. Chin. Sci. Bull. 51, 457–463. doi: 10.1007/s11434-006-0457-130311153

