# Supplementary material for: Seasonal and regional divergence in snow-regulated early-season vegetation productivity on the Tibetan Plateau
Source: Front Plant Sci. 2026 Sep 30;17:1944350. doi: 10.3389/fpls.2026.1944350 (PMC13662066; doi:10.3389/fpls.2026.1944350)
Supplement: Supplementary file 1 [file Table1.docx]

**Figure S1.** Influence pathways of snow depth on spring and summer NPP across the entire Tibetan plateau.

**Figure S2.** Other path coefficients that directly or indirectly regulate spring NPP. Note: In the figure, P represents positive correlation, and N represents negative correlation. TMP represents temperature, SM represents soil moisture, SOS represents the start of the growing season, NPP represents net primary productivity, PRE represents precipitation, and RAD represents solar radiation.

**Figure S3.** Other path coefficients that directly or indirectly regulate summer NPP. Note: In the figure, P represents positive correlation, and N represents negative correlation. TMP represents temperature, SM represents soil moisture, SOS represents the start of the growing season, NPP represents net primary productivity, PRE represents precipitation, and RAD represents solar radiation.

**Figure S4.** The correlation between the snowmelt date (SCM) and soil moisture in spring (a) and summer (b), the beginning of the growing season (c), and snow depth (d).

**Figure S5.** The spatio-temporal variation trend of snow phenology. Note: (a) snow start date (SCS), (b) snow melt date (SCM), (c) snow cover duration days (SCD).

**Figure S6.** Comparison of the absolute correlation coefficients between snow depth and soil moisture in spring and summer.

**Table S1** Parameter settings for NPP based on the MODIS NDVI simulation (2000-2020).

| Code | Vegetation type | E_max | VI_max | VI_min |
| --- | --- | --- | --- | --- |
| 1 | Shrubbery | 0.429 | 0.82 | 0.1 |
| 2 | Cultivated plants | 0.542 | 0.7 | 0.1 |
| 3 | Bare land | 0.542 | 0.7 | 0.1 |
| 4 | Marsh | 0.542 | 0.7 | 0.1 |
| 5 | Coniferous forest | 0.485 | 0.83 | 0.1 |
| 6 | Grass | 0.542 | 0.7 | 0.1 |
| 7 | Meadow | 0.542 | 0.7 | 0.1 |
| 8 | Broad-leaved forest | 0.692 | 0.87 | 0.1 |
| 9 | Steppe | 0.542 | 0.7 | 0.1 |
| 10 | Coniferous and broadleaf trees | 0.475 | 0.85 | 0.1 |
| 11 | Desert | 0.542 | 0.7 | 0.1 |
| 12 | Alpine vegetation | 0.542 | 0.7 | 0.1 |

**Table S2** Parameter settings for NPP based on the extended NDVI simulation (1982-1989).

| Code | Vegetation type | E_max | VI_max | VI_min |
| --- | --- | --- | --- | --- |
| 1 | Evergreen coniferous forest | 0.389 | 0.647 | 0.1 |
| 2 | Evergreen broad-leaf forest | 0.985 | 0.676 | 0.1 |
| 3 | Deciduous coniferous forest | 0.485 | 0.738 | 0.1 |
| 4 | Deciduous broadleaved forest | 0.692 | 0.747 | 0.1 |
| 5 | Mixed forest | 0.475 | 0.743 | 0.1 |
| 6 | Dense shrubbery | 0.429 | 0.636 | 0.1 |
| 7 | Sparse shrub forest | 0.429 | 0.636 | 0.1 |
| 8 | Sparse grassland | 0.542 | 0.634 | 0.1 |
| 9 | Steppe | 0.542 | 0.634 | 0.1 |
| 10 | Tundra | 0.542 | 0.634 | 0.1 |
| 11 | Agriculture and natural vegetation | 0.542 | 0.634 | 0.1 |
| 12 | Snow and ice | 0.542 | 0.634 | 0.1 |
| 13 | Bare land | 0.542 | 0.634 | 0.1 |
| 14 | Water body | 0.542 | 0.634 | 0.1 |
| 15 | Dryland | 0.542 | 0.634 | 0.1 |

**Table S3** Parameter settings for NPP based on the extended NDVI simulation (1990-1999).

| Code | Vegetation type | E_max | VI_max | VI_min |
| --- | --- | --- | --- | --- |
| 1 | Evergreen coniferous forest | 0.389 | 0.647 | 0.1 |
| 2 | Evergreen broad-leaf forest | 0.985 | 0.676 | 0.1 |
| 3 | Deciduous broadleaved | 0.692 | 0.747 | 0.1 |
| 4 | Mixed forest | 0.475 | 0.743 | 0.1 |
| 5 | Dense shrubbery | 0.429 | 0.636 | 0.1 |
| 6 | Steppe | 0.542 | 0.634 | 0.1 |
| 7 | Tundra | 0.542 | 0.634 | 0.1 |
| 8 | Snow and ice | 0.542 | 0.634 | 0.1 |
| 9 | Bare land | 0.542 | 0.634 | 0.1 |
| 10 | Water body | 0.542 | 0.634 | 0.1 |
| 11 | Dryland | 0.542 | 0.634 | 0.1 |

**Table S4** Parameter settings for NPP based on the extended NDVI simulation (2000-2009).

| Code | Vegetation type | E_max | VI_max | VI_min |
| --- | --- | --- | --- | --- |
| 1 | Evergreen coniferous forest | 0.389 | 0.647 | 0.1 |
| 2 | Evergreen broad-leaf forest | 0.985 | 0.676 | 0.1 |
| 3 | Deciduous broadleaved forest | 0.692 | 0.747 | 0.1 |
| 4 | Mixed forest | 0.475 | 0.743 | 0.1 |
| 5 | Dense shrubbery | 0.429 | 0.636 | 0.1 |
| 6 | Sparse shrub forest | 0.429 | 0.636 | 0.1 |
| 7 | Steppe | 0.542 | 0.634 | 0.1 |
| 8 | Agriculture and natural vegetation | 0.542 | 0.634 | 0.1 |
| 9 | Snow and ice | 0.542 | 0.634 | 0.1 |
| 10 | Bare land | 0.542 | 0.634 | 0.1 |
| 11 | Water body | 0.542 | 0.634 | 0.1 |
| 12 | Dryland | 0.542 | 0.634 | 0.1 |

**Table S5** Parameter settings for NPP based on the extended NDVI simulation (2010-2020).

| Code | Vegetation type | E_max | VI_max | VI_min |
| --- | --- | --- | --- | --- |
| 1 | Evergreen coniferous forest | 0.389 | 0.647 | 0.1 |
| 2 | Deciduous coniferous forest | 0.485 | 0.738 | 0.1 |
| 3 | Deciduous broadleaved forest | 0.692 | 0.747 | 0.1 |
| 4 | Mixed forest | 0.475 | 0.743 | 0.1 |
| 5 | Dense shrubbery | 0.429 | 0.636 | 0.1 |
| 6 | Sparse shrub forest | 0.429 | 0.636 | 0.1 |
| 7 | Steppe | 0.542 | 0.634 | 0.1 |
| 8 | Tundra | 0.542 | 0.634 | 0.1 |
| 9 | Agriculture and natural vegetation | 0.542 | 0.634 | 0.1 |
| 10 | Snow and ice | 0.542 | 0.634 | 0.1 |
| 11 | Bare land | 0.542 | 0.634 | 0.1 |
| 12 | Water body | 0.542 | 0.634 | 0.1 |
| 13 | Dryland | 0.542 | 0.634 | 0.1 |
